# Type-*f* thioredoxins have a role in the short-term activation of carbon metabolism and their loss affects growth under short-day conditions in *Arabidopsis thaliana*

**DOI:** 10.1093/jxb/erw017

**Published:** 2016-02-02

**Authors:** Belén Naranjo, Antonio Diaz-Espejo, Marika Lindahl, Francisco Javier Cejudo

**Affiliations:** ^1^Instituto de Bioquímica Vegetal y Fotosíntesis, Universidad de Sevilla and CSIC, Avda Américo Vespucio, 49, 41092-Sevilla, Spain; ^2^Instituto de Recursos Naturales y Agrobiología de Sevilla, CSIC, Avda Reina Mercedes, 10, 41012-Sevilla, Spain

**Keywords:** Carbon assimilation, chloroplast, fructose 1,6 *bis*phosphatase, photosynthesis, redox regulation, thioredoxin.

## Abstract

Redox regulation plays a central role in the adaptation of chloroplast metabolism to light. Extensive biochemical analyses *in vitro* have identified *f*-type thioredoxins (Trxs) as the most important catalysts for light-dependent reduction and activation of the enzymes of the Calvin–Benson cycle. However, the precise function of type *f* Trxs *in vivo* and their impact on plant growth are still poorly known. To address this issue we have generated an *Arabidopsis thaliana* double knock-out mutant, termed *trxf1f2*, devoid of both *f*1 and *f*2 Trxs. Despite the essential function previously proposed for *f*-type Trxs, the visible phenotype of the *trxf1f2* double mutant was virtually indistinguishable from the wild type when grown under a long-day photoperiod. However, the Trx *f*-deficient plants showed growth inhibition under a short-day photoperiod which was not rescued at high light intensity. The absence of *f*-type Trxs led to significantly lower photosynthetic electron transport rates and higher levels of non-photochemical energy quenching. Notably, the Trx *f* null mutant suffered from a shortage of photosystem I electron acceptors and delayed activation of carbon dioxide fixation following a dark–light transition. Two redox-regulated Calvin–Benson cycle enzymes, fructose 1,6-bisphosphatase (FBPase) and Rubisco activase, showed retarded and incomplete reduction in the double mutant upon illumination, compared with wild-type plants. These results show that the function of *f*-type Trxs in the rapid activation of carbon metabolism in response to light is not entirely compensated for by additional plastid redox systems, and suggest that these Trxs have an important role in the light adjustment of photosynthetic metabolism.

## Highlight

*Arabidopsis* plants lacking type-*f* thioredoxins show an impairment of photosynthetic performance indicating specific functions for these thioredoxins not compensated for by other redox systems.These functions only affect growth under short-day conditions.

## Introduction

Chloroplasts are essential for plant life because these organelles perform photosynthesis, the process that allows the conversion of light energy into biomass with the concomitant production of molecular oxygen. In addition, chloroplasts act as sensors of environmental conditions, particularly light quantity and quality, thus playing an important role in harmonizing the growth of plant photosynthetic and non-photosynthetic tissues as well as the adaptation of plants to the environment (Jarvis and López-Juez, 2013). To meet these requirements, chloroplast metabolism needs to respond rapidly to external and internal signals, redox regulation being an important aspect of this adaptability. Redox regulation is a post-translational modification consisting of the dithiol-disulphide interchange of selected and well-conserved cysteine residues of proteins. It is thus a reversible mechanism that allows the rapid regulation of metabolic pathways (Buchanan *et al.*, 2012). Protein disulphide reductases such as thioredoxins (Trxs), small polypeptides of 12–14kDa with a highly conserved active site (WCGPC) and a characteristic structure, the so-called Trx-fold, play a central role in redox regulation. Trxs catalyse the reduction of disulphide bridges in target proteins and, as a consequence, the two cysteine residues at the Trx active site become oxidized as a disulphide bridge. Therefore, for a new catalytic cycle this disulphide needs to be reduced, reducing power being provided by NADPH in a reaction catalysed by NADPH-dependent Trx reductase (NTR). This two-component redox system formed by NTR and Trx is universally distributed in all kinds of organisms from bacteria to fungi, animals, and plants (Jacquot *et al.*, 2009).

In contrast to heterotrophic organisms and non-photosynthetic plant tissues, redox regulation in chloroplasts has unique features. These organelles harbour a complex set of Trxs and Trx-like proteins (Lemaire *et al.*, 2007; Chibani *et al.*, 2009; Meyer *et al.*, 2012; Balsera *et al.*, 2014 ), which are not reduced by NADPH, but by ferredoxin (Fdx) reduced by photosynthetic electron transport in a process catalysed by a Fdx-dependent Trx reductase (FTR) found exclusively in plastids and cyanobacteria (Dai *et al.*, 2007). Therefore, chloroplast redox regulation is mediated by the Fdx/FTR/Trx system and, thus, is dependent on light. Initial biochemical analyses *in vitro* led to the identification of two types of Trxs in chloroplasts, termed *f* and *m*, based on their ability to reduce and activate fructose-1,6-bisphophate phosphatase (FBPase) and NADP-malic dehydrogenase (NADP-MDH), respectively (Wolosiuk *et al.*, 1979). The availability of genome sequences from different plants has uncovered the complex set of chloroplastic Trxs, which in *Arabidopsis thaliana* include two isoforms of *f*-type, four isoforms of *m*-type, two isoforms of the *y*-type, and an *x*-type Trx (Lemaire *et al.*, 2007; Meyer *et al.*, 2012; Balsera *et al.*, 2014). Trxs *f* and *m* were proposed to play a predominant role in the redox regulation of the central biosynthetic pathways, such as the Calvin–Benson cycle, whereas Trxs *x* and *y* show a capacity to reduce peroxiredoxins (Prxs) and so were considered to have an antioxidant function (Collin et al., 2003, 2004). In addition, a novel Trx, type-*z*, was identified in *Arabidopsis*, which is involved in the redox regulation of plastid transcription (Arsova *et al.*, 2010; Schröter *et al.*, 2010; Steiner *et al.*, 2011; Wimmelbacher and Börnke, 2014). Beside these canonical Trxs, several atypical Trxs have been described in the chloroplast. This is the case of HCF164, which is localized in the thylakoid membrane facing the lumen (Motahashi and Hisabori, 2006), and the small family of atypical Trxs identified in *Arabidopsis* termed AtACHTs (for atypical Cys His-rich Trxs) (Dangoor *et al.*, 2009). Finally, different Trx-like proteins were identified in chloroplasts, among which the so-called CDSP32 is the best characterized (Broin *et al.*, 2002).

In addition, chloroplasts harbour an NADPH-dependent redox system based on the activity of a bimodular enzyme consisting of an NTR with a joint Trx domain at the C-terminus, termed NTRC (Serrato *et al.*, 2004; Kirchsteiger *et al.*, 2012). NTRC is able to conjugate both NTR and Trx activities for the efficient reduction of 2-Cys Prxs (Pérez-Ruiz *et al.*, 2006; Moon *et al.*, 2006; Alkhalfioui *et al.*, 2007), thus suggesting an antioxidant function for this enzyme (Puerto-Galán *et al.*, 2013). Additional reports indicate a function of NTRC in the redox regulation of starch synthesis (Michalska *et al.*, 2009; Lepistö *et al.*, 2013) and in different reactions of the biosynthesis of tetrapyrroles (Richter *et al.*, 2013; Pérez-Ruiz *et al.*, 2014). The high affinity of NTRC for NADPH introduces the notion that redox regulation in chloroplasts relies not only on photosynthetically reduced Fdx but also on NADPH which can be produced during the night from sugars by the oxidative pentose phosphate pathway (Spínola *et al.*, 2008; Cejudo *et al.*, 2012).

In parallel with the knowledge of the increasing complexity of the plastidial Trxs, the advance in proteomics has allowed the identification of a large number of putative targets of Trxs (Balmer *et al.*, 2003; Buchanan and Balmer, 2005; Montrichard *et al.*, 2009), which indicates that redox regulation is important for virtually any process taking place in the chloroplast. However, the question of the level of specificity or redundancy of the different Trxs in redox regulation in this organelle is still poorly understood. An *Arabidopsis* knockout mutant deficient in Trx *z* showed a severe phenotype indicating that the function of this Trx in chloroplast transcription is not redundant with other plastidial Trxs (Arsova *et al.*, 2010). By contrast, an *Arabidopsis* knockout mutant lacking Trx *x* shows almost a wild-type phenotype, indicating that this deficiency is compensated for by other plastidial redox systems (Pulido *et al.*, 2010). More uncertain is the specificity of Trxs with several isoforms, such as those of type *m* or *f*, which are considered to play a relevant role in the redox regulation of photosynthetic metabolism. Trx *m*4 is involved in alternative photosynthetic electron transport (Courteille *et al.*, 2013), and the simultaneous deficiency of Trxs *m*1, *m*2, and *m*4 resulted in impaired photosystem II biogenesis (Wang *et al.*, 2013).

Proteomic and biochemical studies *in vitro* have shown the relevant function of type-*f* Trxs in the redox regulation of most of the enzymes of the Calvin–Benson cycle (Michelet *et al.*, 2013). In addition, an *Arabidopsis* mutant lacking Trx *f*1 shows impaired light-dependent reductive activation of ADP-glucose pyrophosphorylase (AGPase) and starch turnover (Thormählen *et al.*, 2013). Surprisingly, despite the key role proposed for type-*f* Trxs in the redox regulation of chloroplast metabolism, the Trx *f*1-deficient mutant shows a wild-type phenotype (Thormählen *et al.*, 2013). Moreover, a double mutant devoid of both Trx *f*1 and Trx *f*2 showed a visible phenotype indistinguishable from wild-type plants (Yoshida *et al.*, 2015), suggesting that other plastidial redox systems are able to compensate for the deficiency of *f*-type Trxs. Therefore, with the aim of establishing the role of type *f* Trxs in chloroplast performance and plant growth, we generated a double knockout mutant of *Arabidopsis* devoid of Trx *f*1 and Trx *f*2, and have performed the analysis of its phenotype under different growth conditions. The double mutant shows no visible phenotype, compared with wild-type plants, when grown under long-day conditions, however, it shows retarded growth under short-day conditions which is not rescued by high light intensity. Analysis of photosynthetic parameters and changes in the redox status of FBPase and Rubisco activase in response to light showed that type-*f* Trxs are required for the rapid reduction of the Calvin–Benson cycle enzymes in response to light, a function not compensated for by other plastidial redox systems.

## Materials and methods

### Growth conditions and plant material

*Arabidopsis thaliana* wild-type (ecotype Columbia) and mutant plants were grown in soil in growth chambers under long-day (16/8h light/dark) or short-day (8/16h light/dark) conditions at 22 °C during the light and 20 °C during the dark periods and a light intensity of 125 μE m^−2^ s^−1^. For experiments addressing the effects of irradiance, plants were grown under short-day conditions at 125, 350, and 950 μE m^−2^ s^−1^ light intensity. A homozygous line, GK-020E05-013161, with a T-DNA insertion in the *TRX f2* gene (see Supplementary Fig. S1 at *JXB* online) from *Arabidopsis*, termed the *trxf2* mutant, was selected by PCR analysis with the oligonucleotides described in Supplementary Table S1. This mutant was manually crossed with the *trxf1* mutant, which was previously reported by Pérez-Ruiz et al. (2014). Seeds resulting from this cross were tested for heterozygosity of the T-DNA insertions in the *TRX f1* and *TRX f2* genes. Plants were then self-crossed and double homozygous lines were identified in the progeny by PCR analysis of genomic DNA using the oligonucleotides described in Supplementary Table S1.

### RNA extraction and qRT-PCR analysis

Total RNA was extracted using the TRIsure RNA extraction reagent (BIOLINE) and cDNA synthesis was performed with 1 μg of total RNA using the Maxima first strand cDNA synthesis kit (Thermo Scientific) according to the manufacturer’s instructions. The content of Trx *f*1 and Trx *f*2 transcripts was determined by real time quantitative PCR (qRT-PCR) with RNA samples extracted from seedlings. qRT-PCR was performed with oligonucleotides shown in Supplementary Table S2 in an IQ5 real-time PCR detection system (Bio-Rad) following a standard thermal profile (95 °C, 3min, 40 cycles of 95 °C for 10s, and 60 °C for 30s). The relative level of each transcript was referred to the level of the *UBIQUITIN* transcript.

### Protein extraction, alkylation assays, and Western blot analysis

For protein extraction, leaves were ground in liquid nitrogen and 10% (v/v) trichloroacetic acid (TCA) was immediately added to quench thiol oxidation. Samples were incubated on ice for 20min and then centrifuged at 16 200 *g* at 4 °C for 10min. The pellets were washed with acetone, resuspended in alkylation buffer (2% SDS, 50mM TRIS–HCl pH 7.8, 2.5% glycerol, and 4M urea) with 10mM methyl-maleimide polyethylene glycol (MM-PEG_24_) and incubated for protein thiol alkylation for 20min at room temperature. Samples were subjected to SDS-PAGE (9.5% polyacrylamide), transferred on to nitrocellulose membranes, and probed with an anti-FBPase antibody which was kindly provided by Dr M Sahrawy, Estación Experimental del Zaidín, Granada, Spain, or with an anti-Rubisco activase antibody which was kindly provided by Dr AR Portis, USDA, Urbana, USA. The anti-Trx *f* antibody was raised by rabbit immunization with purified recombinant Trx *f*1 from *Arabidopsis* at the Servicio de Producción Animal, University of Seville, Spain. This antibody also detected Trx *f*2 although with somewhat lower efficiency (see Supplementary Fig. S2).

### Measurements of chlorophyll *a* fluorescence and P_700_ absorbance

Room temperature chlorophyll fluorescence was measured using a pulse-amplitude modulation fluorimeter (DUAL-PAM-100, Walz, Effeltrich, Germany). The maximum PSII quantum yield, determined as variable fluorescence (*F*_v_) to maximal fluoresence (*F*_m_), *F*_v_/*F*_m_, was measured after the dark adaptation of the plants for 30min and a single saturating pulse of red (635nm) light at 10 000 μE m^−2^ s^−1^ was applied. Induction-recovery curves were performed using red (635nm) actinic light at 75 μE m^−2^ s^−1^ for 8min. Saturating pulses of red light at 10 000 μE m^−2^ s^−1^ intensity and 0.6s duration were applied every 60s and recovery in darkness was recorded for up to 10min. The parameters *Y*(II) and *Y*(NPQ) corresponding to the respective quantum yields of PSII photochemistry and non-photochemical quenching (NPQ) were calculated by the DUAL-PAM-100 software according to the equations in Kramer *et al.* (2004). Relative linear electron transport rates were measured in leaves of pre-illuminated plants by applying stepwise increasing actinic light intensities up to 2 000 μE m^−2^ s^−1^. The redox state of photosystem I P_700_ was monitored by following the changes in absorbance at 830nm versus 875nm using the DUAL-PAM-100. Plants were kept in the dark for 30min and then, to probe the maximum extent of P_700_ oxidation, leaves were illuminated with far red (730nm) light superimposed on the actinic light. Thereafter, absorbance traces were recorded during a 5min illumination with 126 μE m^−2^ s^−1^ red (635nm) actinic light followed by 5min darkness. Saturating pulses of red light at 10 000 μE m^−2^ s^−1^ were applied every 20s. The quantum yields of PSI photochemistry *Y*(I), donor side limitations *Y*(ND), and acceptor side limitations *Y*(NA) were based on saturating pulse analysis and calculated by the DUAL-PAM-100 software.

### Determination of the rate of carbon assimilation *A*_N_

Net CO_2_ assimilation rate (*A*_N_) was measured using an open gas exchange system Li-6400 equipped with the chamber head (Li-6400–40). All measurements were conducted in dark-adapted leaves of short-day grown plants (50 d-old) at 500 μmol mol^−1^ of CO_2_, a constant leaf temperature of 20 °C, and a vapour pressure deficit between leaf and air of below 1 kPa. Before the light, at an intensity of 70 μE m^−2^ s^−1^, was turned on in the leaf chamber, *A*_N_ was recorded for 30min every 5s, then recording continued until equilibrium was reached. Six leaves were measured per line.

### Determination of chlorophylls

Leaf discs were weighed and incubated in 1ml methanol overnight at 4 °C. After extraction, chlorophyll levels were measured spectrophotometrically, as described in Porra *et al.* (1989), and normalized to fresh weight or leaf area. The values were compared with a Tukey Test (Anova) using a confidence interval of 99%.

### Determination of starch content

Starch content was determined in leaves of wild-type and the Trx *f*-deficient mutant grown under long-day conditions harvested before flowering (22–26-d-old), essentially as described in Lin *et al.* (1988). For starch extraction, leaves (100–200mg fresh weight) harvested at the end of the day were ground in liquid nitrogen and washed with 80% (v/v) ethanol in 10mM HEPES pH 7.6, for 2h at 80 °C before being washed with the same solution at room temperature until the tissue was free of pigments. Dry pellets, after centrifugation, were resuspended in 1ml of 0.2N KOH and heated at 100 °C for 30min. After cooling, samples were centrifuged at 17 000 *g* for 10min and the supernatant was adjusted to pH 5.0 with 1N acetic acid. An aliquot of 200 μl of this solution was used to determine the amount of starch using the enzymatic method described by Lin *et al.* (1988). The values were compared with a Tukey Test (Anova) using a confidence interval of 99%.

## Results

### Type-*f* Trxs are dispensable for plant growth

**Fig. 1.**
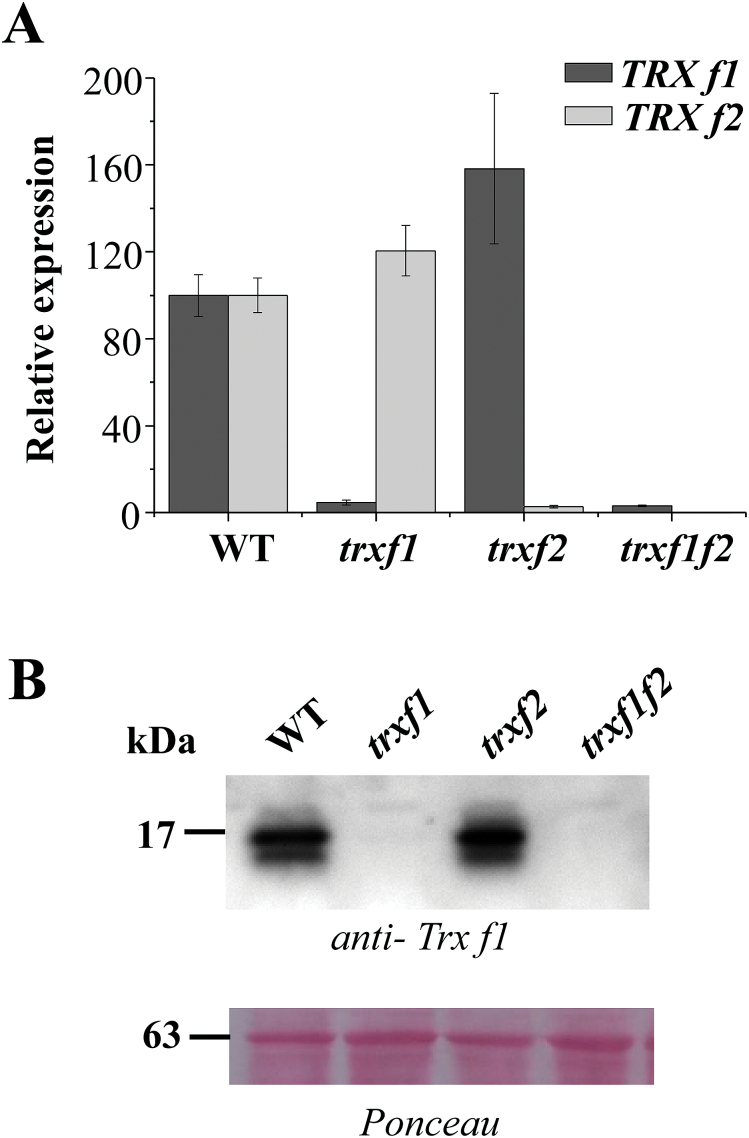
The level of Trx *f*1 and Trx *f*2 transcripts and proteins in wild-type and mutant plants. (A) The content of Trx *f*1 and *f*2 transcripts was determined by qRT-PCR from total RNA, which was extracted from wild-type, *trxf1*, *trxf2,* and *trxf1f2* seedlings. The pairs of oligonucleotides used for cDNA amplification are indicated in Supplementary Table S2. Transcript levels were normalized to *UBIQUITIN* amplification and referred to the level of *TRX f1* and *TRX f2* in wild-type plants. Determinations were performed three times and mean values ±SD are represented. (B) Western blot analysis of the content of type-*f* Trxs in wild-type and mutant plants. Proteins were extracted from leaves (100mg fresh weight) of wild-type, *trxf1*, *trxf2*, and *trxf1f2* plants grown under short-day conditions and subjected to SDS-PAGE (13% polyacrylamide) under reducing conditions, transferred to a nitrocellulose membrane, and probed with an anti-Trx *f*1 antibody. Ponceau staining was used as a loading control. Molecular weight markers in kDa are indicated on the left.

The *Arabidopsis* genome encodes two isoforms of *f*-type Trxs, *f*1 and *f*2. It was recently reported that *Arabidopsis* knockout mutants lacking either Trx *f*1 (Thormählen *et al.*, 2013) or both Trx *f*1 and Trx *f*2 (Yoshida *et al.*, 2015) show a wild-type phenotype with respect to growth and pigmentation. These results suggest that the loss of *f*-type Trxs might be compensated for by other plastidial redox systems. To address this issue in more detail, we have generated a double knockout mutant of *Arabidopsis* devoid of both Trx *f*1 and Trx *f*2 and have analysed its growth under different conditions. To that end, the *Arabidopsis* line (GK-020E05-013161) with a T-DNA insertion at the AT5G16400.1 locus encoding Trx *f*2 was isolated (Supplementary Fig. S1). This line was manually crossed with the Trx *f*1 knockout mutant (SALK_128365.45.75.x) previously reported by our group (Pérez-Ruiz *et al.*, 2014), which was also characterized by Thormählen et al. (2013). Plants homozygous for both T-DNAs were selected by PCR analysis of genomic DNA (Supplementary Fig. S1). The double mutant, here termed *trxf1f2*, was effectively devoid of both Trx *f*1 and Trx *f*2 as shown by the lack of transcripts of the two genes, based on qRT-PCR (Fig. 1A), and the corresponding polypeptides, as shown by Western blots probed with an antibody raised against Trx *f*1 (Fig. 1B), which also cross-reacted with Trx *f*2 (Supplementary Fig. S2). These results confirmed that Trx *f*1 is much more abundant than Trx *f*2 in wild-type plants. In addition, we tested whether the absence of type-*f* Trxs had any effect on the expression of other plastidial redox systems, but only minor differences in the levels of the transcripts of genes encoding NTRC, type-*m* and *x* Trxs were detected (see Supplementary Fig. S3 ).

**Fig. 2.**
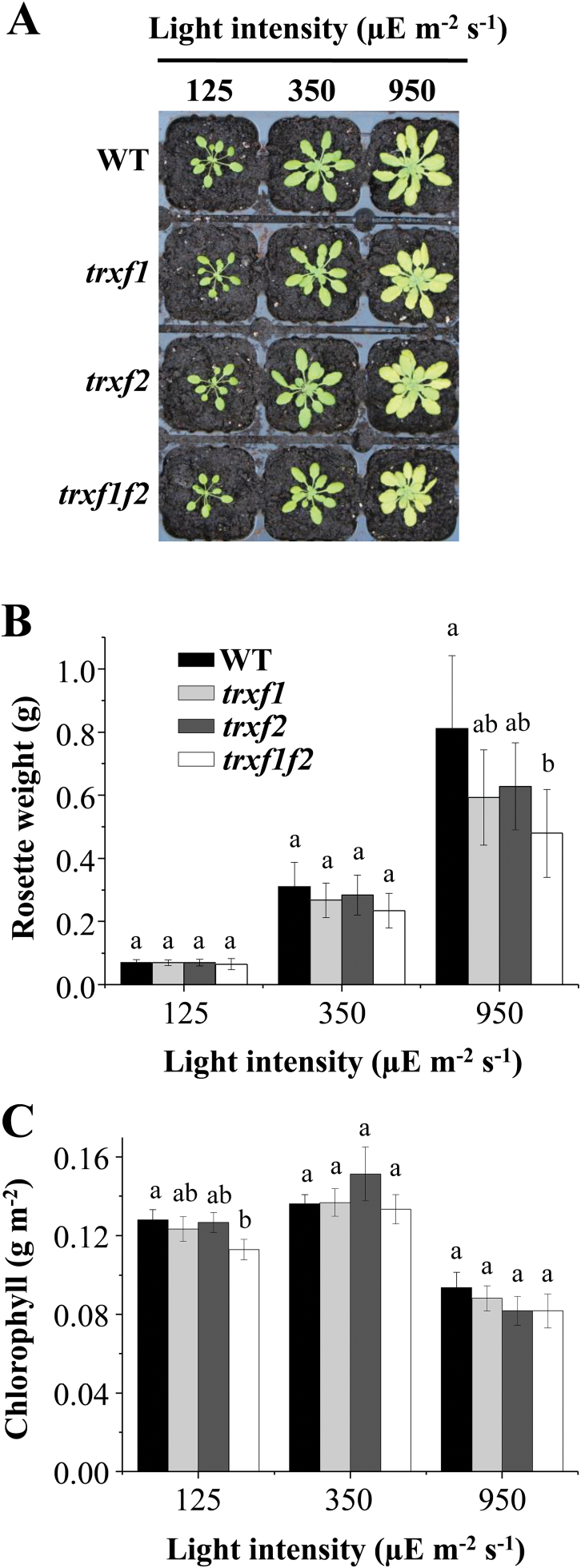
Growth of *Arabidopsis* lines lacking type-*f* Trxs under a long-day photoperiod. (A) Plants of the wild type and mutant lines grown under long-day conditions (16/8h light/dark, light intensity 125 μE m^−2^ s^−1^) for 22 d. (B) The weight of the rosette leaves was determined from nine plants, except for the *trxf1f2* double mutant which was determined from 14 plants. (C) Chlorophyll content was determined from leaf discs (*n*=6) and average values ±SD are represented. Letters indicate significant differences with the Tukey Test and a confidence interval of 99%.

**Fig. 3.**
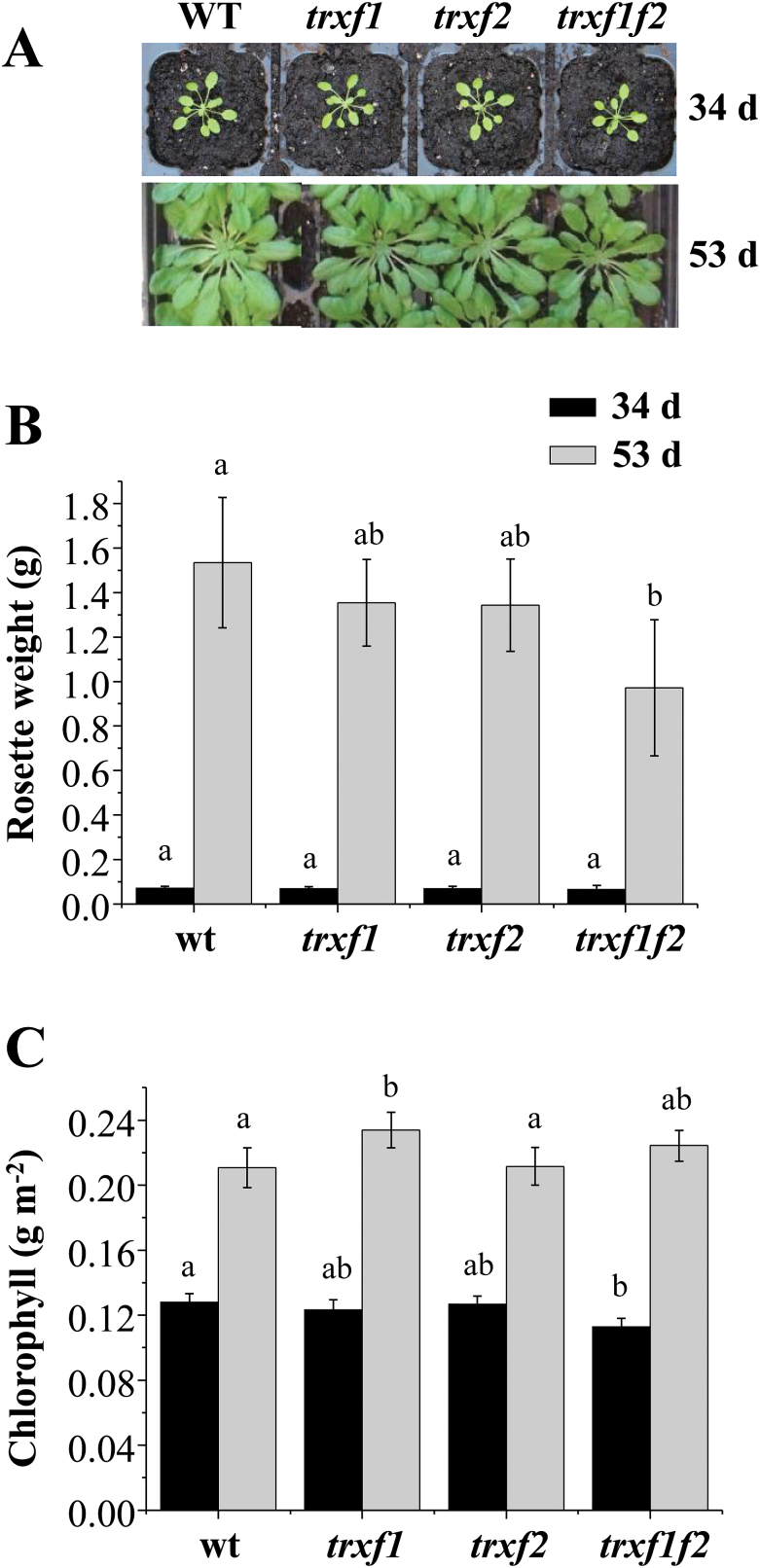
Growth of *Arabidopsis* lines lacking type-*f* Trxs under a short-day photoperiod. (A) Representative plants of the wild type and mutant lines grown under short-day conditions (8/16h light/dark, light intensity 125 μE m^−2^ s^−1^) for 34 d and 53 d. (B) The weight of the rosette leaves was determined from 12 plants (34 d of growth) or seven plants (53 d of growth), except for the *trxf1f2* double mutant which was determined from 14 plants in both cases. (C) Chlorophyll content was determined from leaf discs (*n*=6). Average values ±SD are represented. Letters indicate significant differences with the Tukey Test and a confidence interval of 99%.

**Fig. 4.**
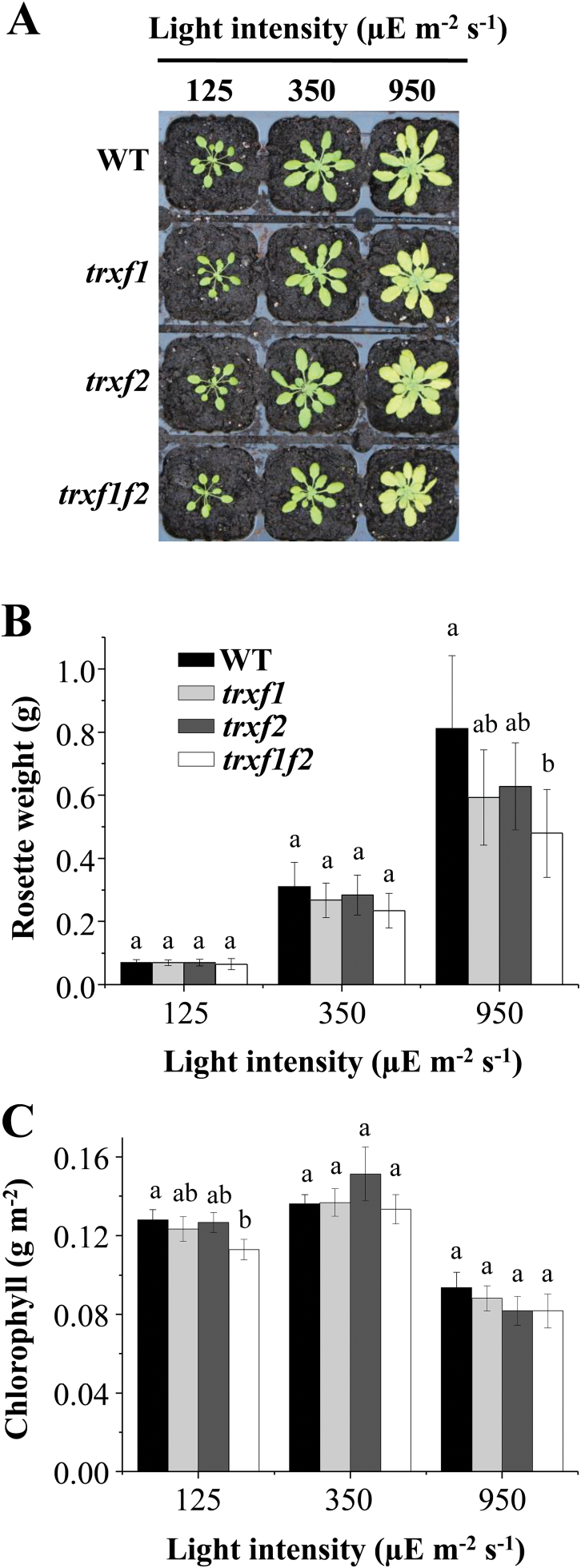
Effect of light intensity on growth of *Arabidopsis* lines lacking type-*f* Trxs under a short-day photoperiod. (A) Representative plants of the wild type and mutant lines grown under short-day conditions (8/16h light/dark) for 34 d at increased light intensities, as indicated. (B) The weight of the rosette leaves from all lines was determined from 12 plants. (C) Chlorophyll content was determined from leaf discs (*n*=6). Average values ±SD are represented. Letters indicate significant differences with the Tukey Test and a confidence interval of 99%.

The *trxf1f2* double mutant, like the single mutants *trxf1* and *trxf2*, showed the wild-type phenotype when grown under the long-day photoperiod (Fig. 2A), as confirmed by the weight of the rosette leaves (Fig. 2B) and leaf chlorophyll content (Fig. 2C) of mature plants immediately before bolting. To analyse in more detail the effect of Trx *f* deficiency on plant growth, the different lines were grown under short-day conditions. Wild-type and Trx *f*-deficient mutants showed an indistinguishable growth rate up to the stage of young rosette leaves (34 d of growth), as determined by fresh weight (Fig. 3A, B). However, after 53 d of growth, the double mutant displayed a significant growth inhibition, as shown by the lower weight of rosette leaves (Fig. 3B), although the chlorophyll content was unaffected (Fig. 3C). Data of the long growth period, 53 d, are only shown for short-day (Fig. 3) and not for long-day conditions (Fig. 2), because the plant developmental stages, adult plants before bolting and advanced leaf senescence, respectively, are not comparable. We then analysed the effect of different light intensities on these mutants grown under short-day conditions. While at 125 and 350 μE m^−2^ s^−1^ light intensity no difference was observed between the wild type and the mutant lines after 34 d of growth, Trx *f*-deficient mutants grown at 950 μE m^−2^ s^−1^ light intensity displayed lower rosette weights than the wild-type plants (Fig. 4A, B). At high light intensity the leaf chlorophyll content was decreased, but no significant differences were observed between wild-type and mutant plants (Fig. 4C). Therefore, despite the central function previously attributed to type *f* Trxs in the redox regulation of chloroplast metabolism, these Trxs are dispensable for plant growth under the long-day photoperiod. Nevertheless, the retarded growth of the *trxf1f2* double mutant under the short-day photoperiod at adult plant stages, even under high light conditions, confirms the light-dependent participation of *f*-type Trxs in chloroplast photosynthetic metabolism.

### Activation of photosynthesis upon a dark–light transition is retarded in plants lacking type *f* Trxs

**Fig. 5.**
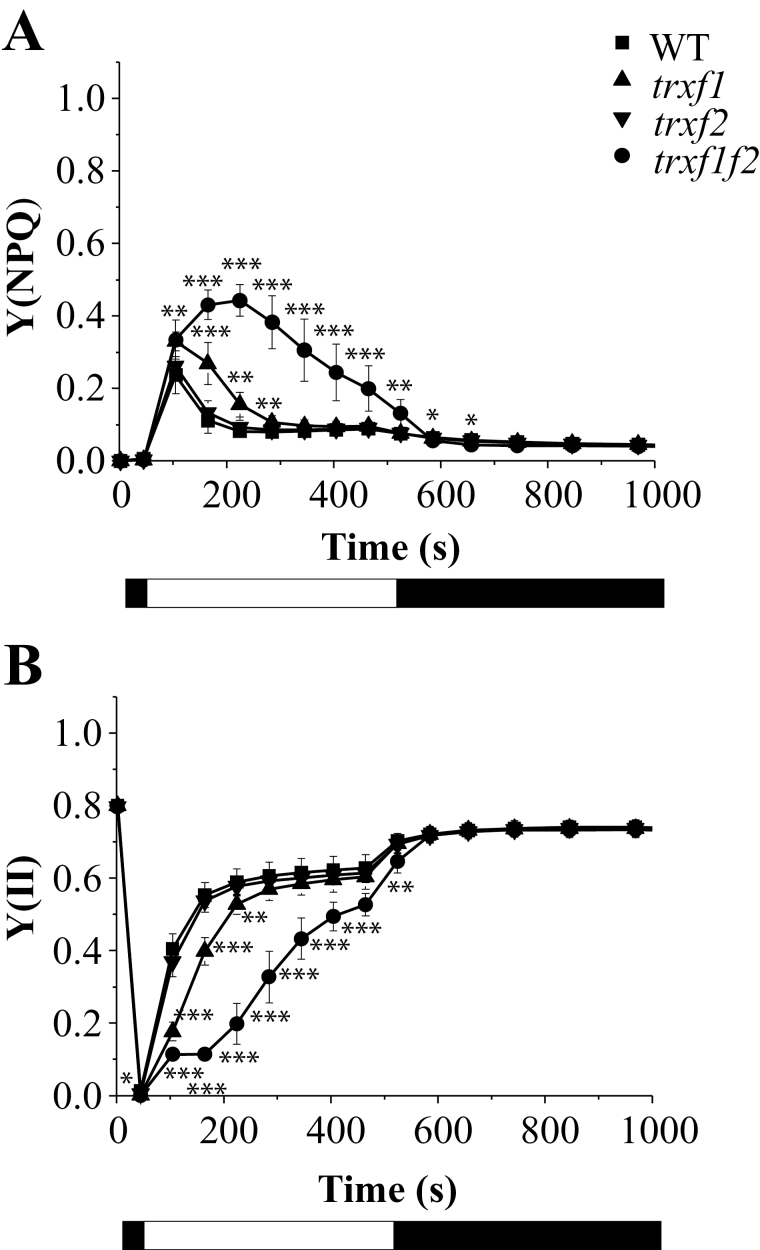
Photosystem II activity in wild type and Trx *f*-deficient mutants. Chlorophyll fluorescence of photosystem II (P_680_) was measured using a pulse-amplitude modulation fluorimeter. After incubation of the plants for 30min in darkness, an induction–recovery curve was performed to determine the quantum yields of non-photochemical quenching, *Y*(NPQ) (A) and photosystem II, *Y*(II) (B). During the 8min induction period, a red (653nm) actinic light at 75 μE m^−2^ s^−1^ intensity was applied. Thereafter, the actinic light was switched off and measurements were continued for another 10min in the dark. Saturation pulses (10 000 μE m^−2^ s^−1^, 0.6s) every 60s were applied. Values are the mean ±SD of 6 plants grown for 53 d under short-day conditions. Light and dark periods are indicated by the white and black bars. Statistical significance (*P <0.05; **P <0.01; ***P <0.001) was determined with Student’s *t* test comparing values for each of the mutant lines with the wild type.

**Fig. 6.**
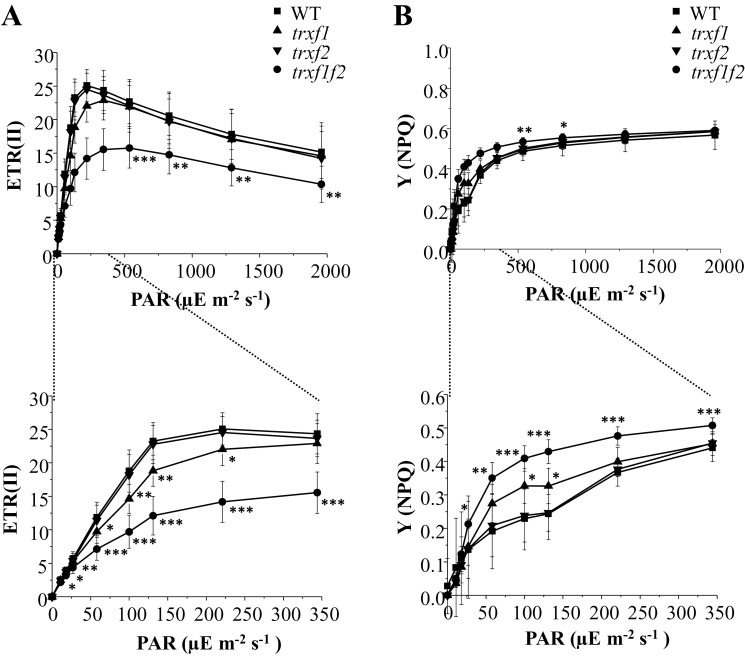
Light-dependent linear photosynthetic electron transport and NPQ in the wild type and Trx *f*-deficient mutants. (A) Relative linear electron transport rates of photosystem II, ETR (II) was measured in pre-illuminated attached leaves of plants grown at 125 μE m^−2^ s^−1^ under short-day conditions. Chlorophyll fluorescence of photosystem II was determined using a pulse-amplitude modulation fluorimeter. Each data point is the mean ±SD of the ETR (II) from eight plants under short-day conditions. PAR (photosynthetically active radiation). (B) Quantum yields of NPQ from the corresponding light-titration curves in (A). Statistical significance (*P <0.05; **P <0.01; ***P <0.001) was determined with Student’s *t* test comparing values for each of the mutant lines with the wild type.

To explore the function of *f*-type Trxs in chloroplast metabolism, different photosynthetic parameters of plants lacking either or both of the Trx *f* enzymes were first examined using chlorophyll fluorescence. The integrity of PSII, determined as the ratio of variable to maximal fluorescence in dark-adapted leaves, was not affected in the single or double *trxf1f2* mutants (Table 1). Non-photochemical quenching (NPQ) is a loss of chlorophyll fluorescence in the light which is not due to photochemistry and reflects adaptation mechanisms regulating the fraction of absorbed light that reaches the PSII reaction centre (Szabó *et al.*, 2005; Baker, 2008). The energy-dependent quenching, *q*E, which involves thermal dissipation of the absorbed light energy, is the main component of NPQ in plants under moderate light intensities and depends on the proton gradient across the thylakoid membrane (Szabó *et al.*, 2005; Baker, 2008; Ruban *et al.*, 2012). Following the onset of actinic light at low or moderate intensity, a brief peak of NPQ is normally observed in wild-type plants due to transient acidification of the thylakoid lumen before activation of photosynthesis (Kalituho *et al.*, 2007). Such an initial peak of NPQ was also found in the *trxf2* mutant and a somewhat broader peak in the *trxf1* mutant (Fig. 5A; see Supplementary Fig. S4). By contrast, the double mutant displayed an extensive NPQ which did not relax completely even after 8min of illumination at 75 μE m^−2^ s^−1^ (Fig. 5A). As a result, the PSII effective quantum yield, *Y*(II), increased more slowly in the light and remained lower in the *trxf1f2* double mutant (Fig. 5B). In agreement with these results, the relative linear photosynthetic electron transport rates were considerably lower in the double mutant at all light intensities examined (Fig. 6A). By contrast, only a small but significant reduction in photosynthetic electron transport rates was observed in the *trxf1* mutant, whereas the *trxf2* mutant displayed wild-type rates (Fig. 6A). Notably, the yields of NPQ were higher in *trxf1f2* plants, particularly at low light intensities (Fig. 6B). These measurements were performed using plants grown under short-day conditions and very similar results were obtained when these photosynthetic parameters were analysed in plants grown under the long-day photoperiod (see Supplementary Figs S5 and S6).

**Table 1.** *F*_v_/*F*_m_ and kinetics of response of net CO_2_ assimilation rate to light

**Genotype**	**WT**	***trxf1***	***trxf2***	***trxf1f2***
*F*_v_/*F*_m_	0.81±0.01	0.82±0.01	0.81±0.01	0.82±0.01
*t*_1/2_ (s)	106.0±2.3 a	187.9±7.5 b	134.3±9.5 a	237.2±10.1 c

The maximum PSII quantum yield was determined as variable fluorescence (*F*_v_) to maximal fluorescence (*F*_m_), *F*_v_/*F*_m_, in dark-adapted leaves of plants grown under short-day conditions. The *F*_v_/*F*_m_ values (±SD) are the average of 12 measurements. *t*_1/2_ represents the time to achieve 50% of the final rate of CO_2_ assimilation, as shown in the data of Supplementary Fig. S5. Data presented are the means (±SE; *n*=6). The differences between mutants and the wild type, when significant, are indicated by different letters (*P* <0.01; Anova, Tukey test).

**Fig. 7.**
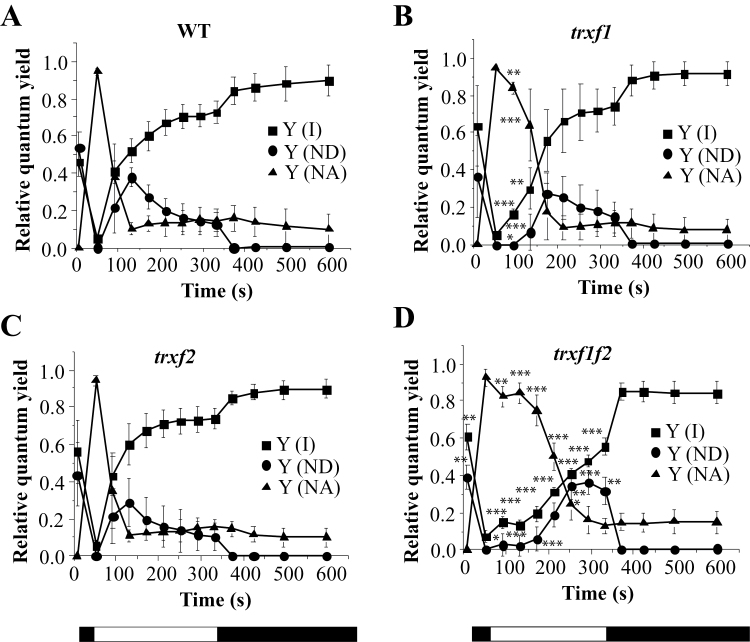
Comparative analysis of photosystem I activity in the wild type and Trx *f*-deficient mutants. The absorbance of oxidized phtotosystem I (P_700_) at 830nm was measured using a pulse-amplitude modulation fluorimeter. The redox state of photosystem I (P_700_) was inferred from changes in this absorbance and monitored for 10min. After 30min dark adaptation (maximum P_700_ reduced), leaves were illuminated with far-red light superimposed on actinic red light (126 μE m^−2^ s^−1^, 635nm) to achieve the maximum oxidation of P_700_ and, subsequently, the actinic light was kept on for 5min followed by another 5min of darkness. White light saturation pulses (10 000 μE m^−2^ s^−1^, 0.6s) were applied every 20s. The quantum yields of PSI *Y*(I), donor side limitations *Y*(ND), and acceptor side limitations *Y*(NA) are based on saturating pulse analysis. Data were collected from six plants grown under short-day conditions and mean ±SD are represented. Statistical significance (*P <0.05; **P <0.01; ***P <0.001) was determined with Student’s *t* test comparing values for each of the mutant lines (B, C, D) with the wild type (A).

The effect of the type *f* Trxs on the control of energy quenching and photosynthetic yield might be through direct interaction with the photosynthetic apparatus or an indirect consequence of limited biosynthesis and demand for ATP, leading to lower luminal pH. The latter case should be revealed by a lower rate of consumption of electrons from photosynthetic electron transport. Therefore, we measured the PSI quantum yield, *Y*(I), based on P_700_ absorbance changes, in mutant and wild-type plants (Fig. 7; see Supplementary Fig. S7). Indeed, the double mutant had a lower PSI activity due to prolonged limitations on the acceptor side, *Y*(NA), after turning on the light (Fig. 7D). This indicates retardation of the activation of the biosynthetic processes which consume reducing equivalents derived from photosynthetic electron transport.

The response of the rate of CO_2_ fixation (*A*_N_) to illumination was more retarded in the *trxf1* than in the *trxf2* single mutant while the *trxf1f2* double mutant displayed even slower kinetics (Table 1; see Supplementary Fig. S8). The half-time for reaching the maximal CO_2_ fixation rate after turning on the light was more than twice as long in the double mutant compared with wild-type plants (Table 1). These results demonstrate the function of *f*-type Trxs in the rapid response of the chloroplast assimilatory metabolism to light.

### Light-dependent reduction of FBPase and Rubisco activase is impaired in the *trxf1f2* double mutant

**Fig. 8.**
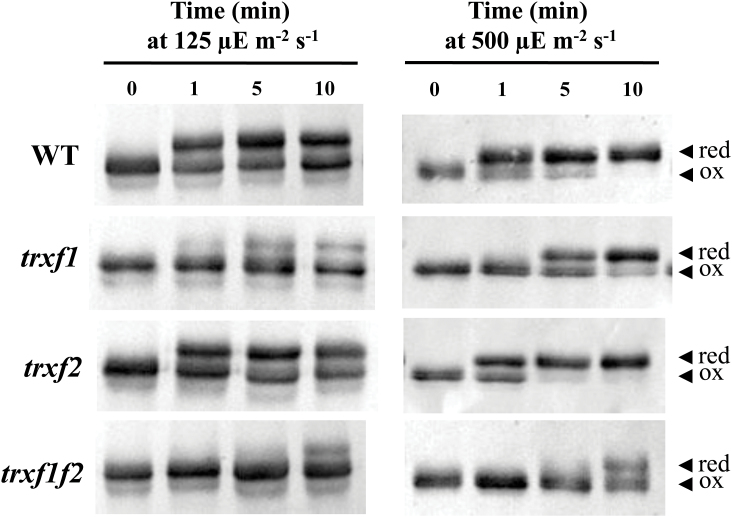
*In vivo* redox status of FBPase in response to light in the wild type and Trx *f* mutant lines. The redox status of FBPase from the different lines under analysis, as indicated on the left, was determined in leaf extracts from plants grown under short-day conditions and harvested at the end of the dark period (time 0), and after 1, 5, and 10min of illumination at the indicated light intensities. Total leaf proteins were extracted in the presence of 10% TCA and protein thiols were alkylated with 10mM MM-PEG_24_. Proteins were resolved in SDS-PAGE (9.5% polyacrylamide) under non-reducing conditions, transferred to nitrocellulose filters, and probed with an anti-FBPase antibody; red, reduced; ox, oxidized.

**Fig. 9.**
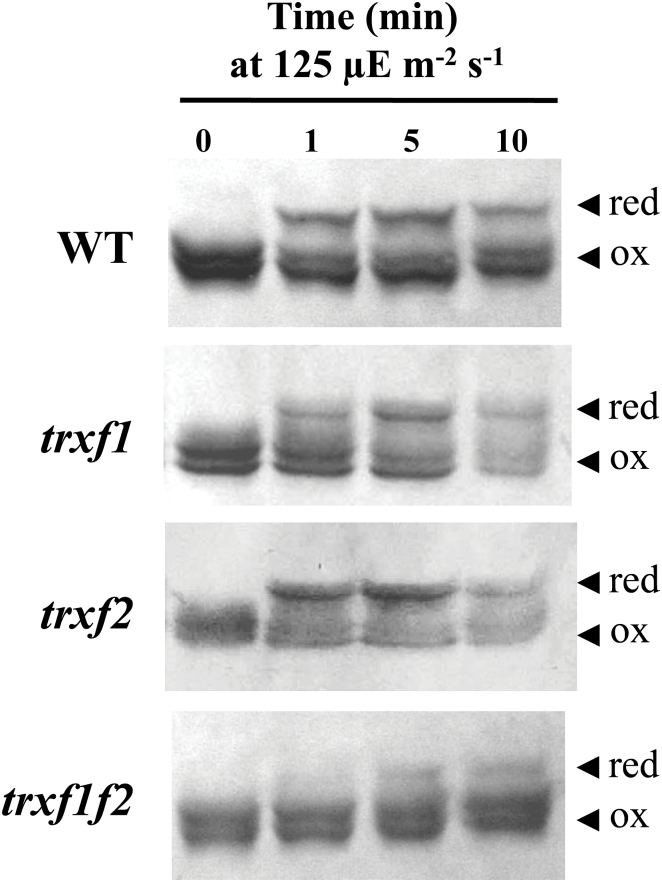
*In vivo* redox status of Rubisco activase in response to light in the wild type and Trx *f* mutant lines. The redox status of Rubisco activase from the different lines under analysis, as indicated on the left, was determined in leaf extracts from plants grown under short-day conditions and harvested at the end of the dark period (time 0), and after 1, 5, and 10min of illumination. Total leaf proteins were extracted in the presence of 10% TCA and protein thiols were alkylated with 10mM MM-PEG_24_. Samples were fractionated in SDS-PAGE (9.5% polyacrylamide) under non-reducing conditions, transferred to nitrocellulose filters, and probed with an anti-Rubisco activase antibody; red, reduced; ox, oxidized.

There is extensive evidence *in vitro* supporting the relevant function of *f*-type Trxs in light-dependent reductive activation of different chloroplast enzymes including those of the Calvin–Benson cycle (recently reviewed by Michelet *et al.*, 2013). To analyse further the function of *f*-type Trxs *in vivo*, we examined the change of the redox status of two well-known *in vitro* targets of type *f* Trxs, FBPase and Rubisco activase, in response to light in the mutant lines. To this end, samples were taken at the end of the night period from plants that had been grown at a light intensity of 125 μE m^−2^ s^−1^ and then subjected to illumination with the same or higher (500 μE m^−2^ s^−1^) light intensity. Short-term changes of the redox status of these enzymes were analysed with the aid of the thiol-alkylating agent methyl maleimide-polyethylene glycol_24_ (MM-PEG_24_), which adds 1.24kDa per thiol group, thus producing a shift in the electrophoretic mobility of the labelled proteins that reflects their redox status. In dark-adapted plants FBPase was detected as a single band indicating that, as expected, the enzyme was fully oxidized under these conditions (Fig. 8). In wild-type plants, FBPase becomes rapidly reduced in response to light within seconds after illumination (Fig. 8). The level of reduction of FBPase proved to be dependent on light intensity since the enzyme became fully reduced after 10min at 500 μE m^−2^ s^−1^, whereas the growth light intensity of 125 μE m^−2^ s^−1^ only promoted a partial reduction, approximately 50%, of the enzyme (Fig. 8). While the deficiency of Trx *f*2 exerted almost no effect on FBPase reduction in response to light, the deficiency of Trx *f*1 significantly impaired the level of reduction of the enzyme at both light intensities (Fig. 8). The *trxf1f2* double mutant showed not only a pronounced delay of FBPase reduction in response to light but also a lower level of reduction of the enzyme after 10min of illumination, even at the higher light intensity tested here (Fig. 8). A similar pattern of reduction in response to light was observed for another prominent redox-regulated enzyme of the Calvin–Benson cycle, Rubisco activase (Fig. 9). In dark-adapted samples Rubisco activase was detected as a double band corresponding to isoforms of 46 and 43kDa, respectively, due to alternative splicing. The long isoform has a C-terminal extension that includes two cysteines which may form an intramolecular disulphide that renders the enzyme inactive (Zhang and Portis, 1999).

**Fig. 10.**
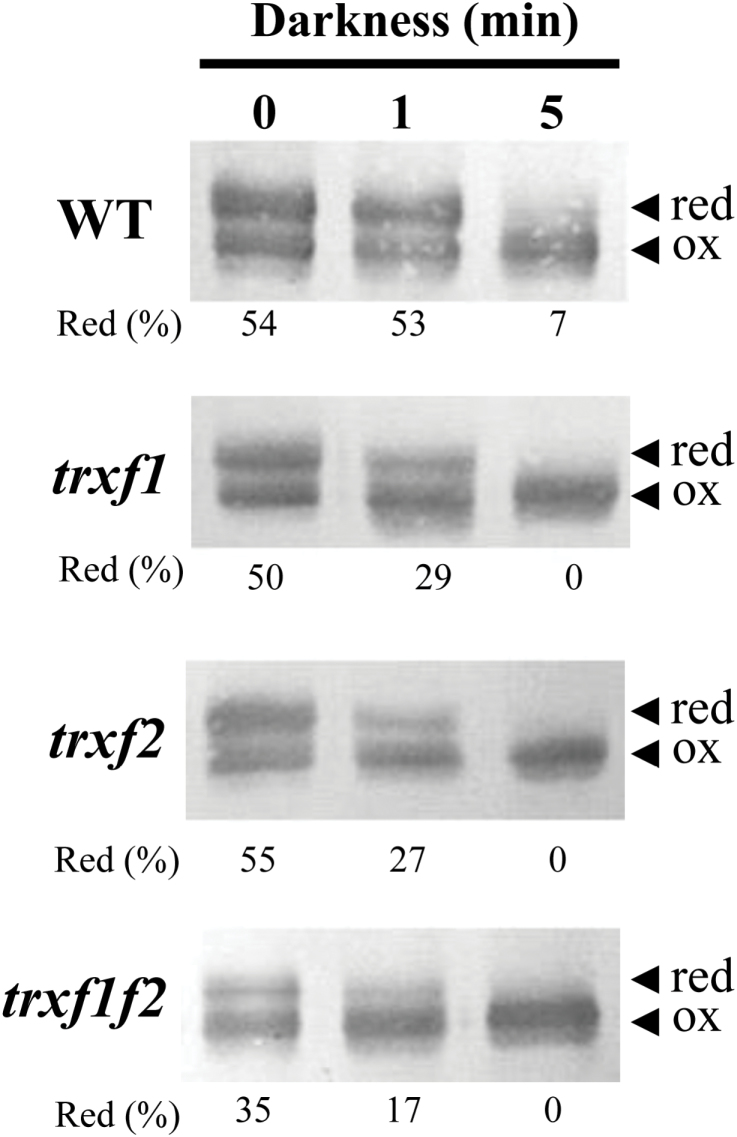
Re-oxidation of FBPase in response to darkness. The redox status of FBPase from the different lines under analysis, as indicated on the left, was determined in leaf extracts from plants grown under short-day conditions and harvested at the end of the light period (time 0), and after 1min and 5min of darkness. Total leaf proteins were extracted in the presence of 10% TCA and protein thiols were alkylated with 10mM MM(PEG)_24_. Proteins were resolved in SDS-PAGE (9.5% polyacrylamide) under non-reducing conditions, transferred to nitrocellulose filters, and probed with an anti-FBPase antibody; red, reduced; ox, oxidized. Band intensities were quantified and the percentage of reduced FBPase is indicated.

**Fig. 11.**
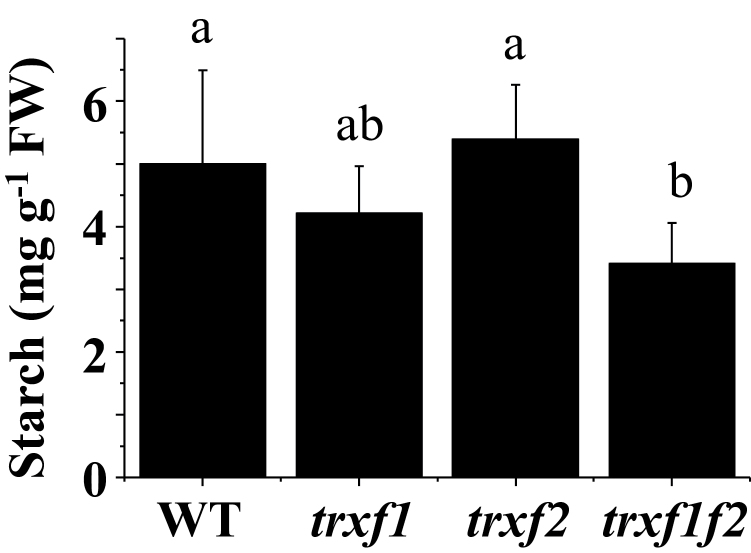
The content of starch in leaves of the wild type and Trx *f* mutant lines. Starch content was determined at the end of the day in leaves from plants grown under long-day conditions for 22–26 d. Data are the mean ±SD of five replicates from two different experiments (with a total of three starch extractions). Letters indicate significant differences with the Tukey Test and a confidence interval of 99%.

Quantification of the reduction of FBPase and Rubisco activase from a series of experiments (such as those presented in Figs 8 and 9), revealed that the levels of reduction of both enzymes remained at 50% of the wild-type level in the *trxf1f2* double mutant after 10min illumination at the growth light intensity (see Supplementary Fig. S9A, B). Furthermore, we analysed the change of the redox status of FBPase following a light–dark transition. Re-oxidation of FBPase in the dark was faster in all three Trx *f* deficient mutants than in wild-type plants (Fig. 10). However, after 5min darkness, FBPase was also nearly completely oxidized in the wild type (Fig. 10). Finally, we compared the starch content in leaves which was significantly lower in the *trxf1f2* double mutant than in the wild type and the single mutants (Fig. 11).

## Discussion

Following the demonstration of the function of Trxs in the light-dependent regulation of chloroplast photosynthetic metabolism (Wolosiuk and Buchanan, 1976), extensive biochemical and proteomic work has shown the central role of *f*-type Trxs in the reductive activation of most of the Calvin–Benson cycle enzymes. In this regard, the findings that overexpression of Trx *f*, but not Trx *m*, enhanced starch accumulation and increased the content of sugars in tobacco leaves (Sanz-Barrio *et al.*, 2013) lend further support to the idea that, among the complex set of plastidial Trxs, those of type *f* play a key role in the redox regulation of carbon metabolism. However, *Arabidopsis* knockout mutants for Trx *f*1, which accounts for up to 95% of the total *f*-type Trxs in this plant, showed impairment of AGPase redox regulation and leaf diurnal starch turnover alterations, yet the visible phenotype of these mutants was indistinguishable from the wild type (Thormählen *et al.*, 2013). Moreover, double mutants lacking both Trx *f*1 and *f*2 were also indistinguishable from wild type plants (Yoshida *et al.*, 2015). Thus, the lack of *f*-type Trxs seems not to affect plant growth despite the relevant function proposed for these Trxs in the redox regulation of chloroplast metabolism based on biochemical analyses. These results suggest that additional redox systems may compensate for the function of *f*-type Trxs being sufficient to support the redox regulation that allows plant growth.

To address this issue, in the present work we have generated a double mutant of *Arabidopsis*, here termed *trxf1f2*, lacking both Trx *f*1 and Trx *f*2. Both qRT-PCR and Western blot analyses (Fig. 1A, B) confirmed that the *trxf1f2* double mutant is a null mutant devoid of *f*-type Trxs. Western blot analysis of leaf extracts from wild-type plants detected a double band, which might correspond to *f*1 and *f*2 Trxs. However, this possibility was ruled out because the double band was detected in the *trxf2* mutant but not in the *trxf1* mutant. Thus, the double band is most probably indicative of a post-translational modification, the nature of which is still unknown. Under long-day conditions the growth of the single mutants, *trxf1* and *trxf2*, was indistinguishable from that of wild-type plants, consistent with previous studies on Trx *f*1-deficient mutants (Thormählen *et al.*, 2013). Similarly, the double mutant displayed the wild-type phenotype when grown under long-day conditions, as shown for type-*f* null mutants recently reported (Yoshida *et al.*, 2015). However, under short-day conditions, single mutants *trxf1* and *trxf2* showed slightly retarded growth (Fig. 3B), in agreement with the behaviour of the Trx *f*1-deficient mutant, which shows retarded growth under short-days, but not under the long-day photoperiod (Thormälen et al., 2015). Adult plants of the *trxf1f2* double mutant showed retarded growth (Fig. 3) which was observed only at later stages of development, indicating that the effect of the deficiency of *f*-type Trxs reported here (lower efficiency of photosynthetic parameters, impaired reduction of Calvin–Benson enzymes, lower starch content) requires time to affect plant growth. Most likely, the delayed growth of the trx *f*-deficient mutant under short-day conditions is not attributable to short-term kinetics of activation, but rather to the lower final level of reduction of enzymes, such as FBPase and Rubisco activase, which can be seen after 10min of illumination (Figs 8, 9) and also at the end of the day (Fig. 10). Moreover, high light intensity (950 μE m^−2^ s^−1^), which caused the decrease in chlorophyll content of both the wild type and mutant lines (Fig. 4C), resulted in retarded growth not only of the double *trxf1f2* mutant but also of the single mutants after 34 d of growth (Fig. 4B). Therefore, deficiency of *f*-type Trxs affects the plant’s response to high light suggesting the participation of these Trxs in plant adaptation to light intensities that might produce oxidative stress. Therefore, despite the central function attributed to type *f* Trxs in chloroplast redox regulation based on biochemical *in vitro* analyses, the *in vivo* approach reported here shows that these Trxs are dispensable for plant growth at least under the standard long-day conditions performed in this study. It should be noted, however, that conditions that may cause light limitation, such as growth under a short-day photoperiod, have a negative effect on the growth rate of Trx *f*-deficient mutants. Moreover, higher light intensity failed to stimulate the growth of mutant plants to the extent that it was stimulated in wild-type plants (Fig. 4), indicating the relevant function of type-*f* Trxs is for plant adaptation to varying light conditions.

The genes encoding Trx *f*1 and Trx *f*2 isoforms are subject to different regulation in *Arabidopsis*. The expression of the *TRX f1* gene responds to light, whereas the *TRX f2* gene is under circadian control (Barajas-López *et al.*, 2011). The differential pattern of expression of these genes might indicate different functions for the respective Trx *f* isoforms. However, all the photosynthetic parameters analysed in this study, such as photosynthetic electron transport and response to illumination of the rate of CO_2_ fixation (*A*_N_), were more affected in the *trxf1* than in the *trxf2* mutant, while the double mutant showed an additive effect. Most probably, these results reflect the higher content of Trx *f*1 in wild-type plants (Fig. 1B) and support a redundant function for both *f*-type Trxs in *Arabidopsis*.

A relevant question concerning chloroplast redox regulation is the level of redundancy or specificity among the many different types of Trx in this organelle. This issue is currently being addressed with the aid of mutants or transgenic plants with altered levels of the different plastidial Trxs. In this regard, the redox status *in vivo* of the Mg chelatase CHLI subunit from pea plants was affected by the simultaneous silencing of the *TRX f* and *TRX m* genes, but not by the silencing of the *TRX f* gene alone (Luo *et al.*, 2012), showing the compensatory effect of Trx *m* on the regulation of this enzyme of the chlorophyll biosynthesis pathway. Therefore, one might assume that *m*-type Trxs could substitute for *f*-type Trxs in the light-dependent redox regulation of the Calvin–Benson cycle enzymes which is in line with the relevant role proposed for *m*-type Trxs in the redox regulation of these enzymes (Okegawa and Motohashi, 2015). However, comparative analyses *in vitro* of a large number of the *Arabidopsis* plastid Trxs (*f*1, *f*2, *m*1, *m*2, *m*3, *m*4, *x*, *y*1, and *y*2) confirmed that only the *f*-type Trxs are capable of activating FBPase using recombinant enzymes (Collin et al., 2003, 2004). The relevant function of *f*-type Trxs in the redox regulation of chloroplast enzymes was further confirmed by *in vivo* studies showing the incomplete reduction of FBPase in mutant plants devoid of Trx *f*1 (Thormählen *et al.*, 2015) and the double mutant devoid of both Trx *f*1 and Trx *f*2 (Yoshida *et al.*, 2015). In addition, purified Rubisco activase has been shown to be readily reduced and activated by spinach Trx *f*, whereas spinach Trx *m* is unable to reduce this enzyme (Zhang and Portis, 1999). In line with the proposed central function of *f*-type Trxs in light-dependent redox regulation of the Calvin–Benson cycle enzymes, our studies *in vivo* show that reduction of both FBPase and Rubisco activase upon illumination is impaired in the *trxf1f2* knockout mutant (Figs 8, 9; Supplementary Fig. S9). Nevertheless, despite the complete absence of Trx *f*, both enzymes become partially reduced during illumination. These results suggest that the *in vitro* studies showing the predominant function of *f*-type Trxs in the redox regulation of the Calvin–Benson cycle enzymes cannot be extrapolated to the physiological situation and show that the double mutant relies on alternative system(s) capable of limited reductive activation of these enzymes in response to light. In this regard, it is worth mentioning that mutant plants devoid of NTRC, an alternative chloroplast redox system, show an incomplete level of FBPase reduction, this effect being even more dramatic in the *ntrc-trxf1* double mutant (Thormählen *et al.*, 2015). Therefore, light-dependent redox regulation of Calvin–Benson cycle enzymes, such as FBPase, seems to be the result of the action of different redox systems including type-*f* Trxs (Fig. 8, this work; Thormählen *et al.*, 2015; Yoshida *et al.*, 2015), type *m* Trxs (Okegawa and Motohashi, 2015), and NTRC (Thormählen *et al.*, 2015). The finding that NTRC and Trx *f*1 act in concert (Thormählen *et al.*, 2015) indicates the existence of cross-talk among these redox systems.

The light-dependent reduction of FBPase and Rubisco activase show very similar patterns in wild-type plants on the one hand and in Trx *f*-deficient plants on the other (Figs 8, 9; Supplementary Fig. S9), suggesting common regulatory mechanisms for the two enzymes. This may be significant since both enzymes are involved in the pathway of CO_2_ fixation in the chloroplast. It should be noted that plant chloroplasts contain two types of FBPase, termed FBPase I and II, but only one of them, FBPase I, is redox-regulated (Serrato *et al.*, 2009). Our results show that, upon illumination with a higher light intensity, 500 μE m^−2^s^−1^, FBPase becomes fully reduced (Fig. 8) indicating that the redox-insensitive form, FBPase II, is present in minor amounts in chloroplasts, in agreement with previous results (Rojas-González *et al.*, 2015).

An interesting observation regarding the redox state of FBPase and Rubisco activase is that these enzymes do not become fully reduced under the growth light intensity even in wild-type plants. This is in agreement with recent reports (Yoshida *et al.* 2014, 2015) showing that *Arabidopsis* plants display only partial reduction of the FBPase when illuminated at low light intensity. However, we observed the complete reduction of FBPase in wild-type plants after 10min illumination at 500 μE m^−2^ s^−1^ light intensity, while the *trxf1f2* double mutant showed a partial reduction of the enzyme (Fig. 8). A remarkable implication of these results is that plants that have been adapted to low light conditions have a significant pool of inactive, not fully reduced, enzymes. A possible advantage of this seemingly wasteful synthesis of excess Calvin–Benson cycle enzymes is that, after a sudden increase in photon flux leading to higher rates of photosynthetic electron transport, the ATP and NADPH generated would immediately be utilized for carbon dioxide fixation and, thus, would not be a problem of light stress. Obviously, a prerequisite for this to occur is that there are sufficiently high amounts of *f*-type Trxs to catalyse the reductive activation of these enzymes. In addition, we have analysed the changes of redox status of FBPase in light–dark transitions. Re-oxidation of FBPase in response to darkness is again a very rapid process which is essentially completed in 5min in wild-type plants (Fig. 10). The fact that re-oxidation is faster in the mutants could indicate that, until exhausted, a pool of reduced *f*-type Trxs continues to catalyse reduction in the dark.

Light-harvesting efficiency and photosynthetic electron transport are highly sensitive to changes in carbon assimilation. For example, sub-atmospheric levels of CO_2_, which result in limited regeneration of ADP and NADP^+^, lead to elevated NPQ and decreased effective PSII quantum yield at low or moderate light intensities (Kramer *et al.*, 2004). Similarly, inhibition of the Calvin–Benson cycle enzymes *in vivo* by iodoacetamide leads to higher NPQ and slower linear photosynthetic electron transport (Joliot and Alric, 2013). Notably, the *q*E component of NPQ ensures safe dissipation of the light energy absorbed and prevents excess excitation of PSII (Szabó *et al.*, 2005; Baker, 2008; Ruban *et al.*, 2012). Such a negative feedback control that arises from hampered CO_2_ fixation is the most likely explanation for the results concerning the activities of PSII and PSI obtained from the *trxf1f2* mutant plants using chlorophyll fluorescence and P_700_ absorbance. Hence, despite normal maximum PSII quantum efficiency, *F*_v_/*F*_m_, the linear photosynthetic electron transport rate is significantly decreased in the double *trxf1f2* knockout mutant compared with the wild type, particularly at lower light intensities. At 100–150 μE m^−2^ s^−1^, which corresponds to growth light intensity, the electron transport in the double mutant was only half that in wild-type plants (Fig. 6A) and the yield of NPQ was twice as high (Fig. 6B).

Although in plants devoid of *f*-type Trxs the response of the rate of carbon assimilation (*A*_N_) to light was delayed (Table 1) these plants still reached carbon assimilation rates similar to those of the wild type (Supplementary Fig. S8). However, the content of starch was diminished in the *trxf1f2* mutant (Fig. 11) which is in line with the impaired activation of the AGPase of the *trxf1* single mutant (Thormählen *et al.*, 2013). Nevertheless, the leaf starch accumulated during the day under a long-day light regime seems sufficient for the correspondingly short night and to support wild-type growth rates (Fig. 2).

## Conclusion

In summary, the *in vivo* approach undertaken in this work identifies the impact of type *f* Trxs on photosynthetic performance in *Arabidopsis*, such as the kinetics of activation of carbon assimilation, the redox status of Calvin–Benson cycle enzymes upon a dark–light transition, and the control of photosynthetic electron transport. The fact that these parameters were impaired in plants devoid of type *f* Trxs indicates that the functions of these Trxs are specific and are not compensated for by other Trxs or chloroplast redox systems. On the other hand, FBPase and Rubisco activase showed a significant level of reduction upon dark–light transition in the *trxf1f2* double mutant indicating that additional chloroplast redox systems participate in light-dependent reduction of these Calvin–Benson cycle enzymes. This stands out against the well-established notion, based on biochemical *in vitro* analyses, of the almost exclusive role attributed to type *f* Trxs in the redox regulation of these enzymes. Surprisingly, the growth of plants devoid of type *f* Trxs is indistinguishable from wild-type plants when grown under standard conditions with a long-day photoperiod, indicating that the function of type *f* Trxs are dispensable for growth.

## Supplementary data

Supplementary data can be found at *JXB* online.

Table S1. Sequence of oligonucleotides used for genotype analyses.

Table S2. Sequence of oligonucleotides used for gene expression analyses.

Figure S1. Genotype of *Trx f*-deficient mutants.

Figure S2. Cross-reaction of the anti Trx *f*1 antibody with Trx *f*1 and Trx *f*2.

Figure S3. Level of *NTRC*, *m*- and *x*-type *TRX* gene transcripts in Trx *f*-deficient mutants.

Figure S4. Chlorophyll *a* fluorescence of PSII.

Figure S5. Photosystem II activity in wild type and Trx *f* deficient mutants grown under long-day conditions.

Figure S6. Light-dependent linear photosynthetic electron transport and NPQ in wild type and Trx *f* deficient mutants grown under long-day conditions.

Figure S7. Absorbance of the oxidized form of PSI.

Figure S8. Net CO_2_ assimilation rate (*A*_N_) in the wild type and Trx *f* deficient mutants.

Figure S9. Light-dependent reduction FBPase and Rubisco activase.

Supplementary Data
