# Supplementary material for: Type-f thioredoxins have a role in the short-term activation of carbon metabolism and their loss affects growth under short-day conditions in Arabidopsis thaliana
Source: J Exp Bot. 2016 Feb 2;67(6):1951–64. doi: 10.1093/jxb/erw017 (PMC4783373; doi:10.1093/jxb/erw017)
Supplement: Supplementary Data [file supp_67_6_1951__index.html]

Type-f thioredoxins have a role in the short-term activation of carbon metabolism and their loss affects growth under short-day conditions in Arabidopsis thaliana — Type-f thioredoxins have a role in the short-term activation of carbon metabolism and their loss affects growth under short-day conditions in Arabidopsis thaliana — Supplementary Data 

# Type-*f* thioredoxins have a role in the short-term activation of carbon metabolism and their loss affects growth under short-day conditions in *Arabidopsis thaliana*

## Supplementary Data

Data files

- supplementary\_tables\_S1\_S2\_figures\_S1\_S9.pdf - Supplementary Data
