# Supplementary material for: Type-f thioredoxins have a role in the short-term activation of carbon metabolism and their loss affects growth under short-day conditions in Arabidopsis thaliana
Source: J Exp Bot. 2016 Feb 2;67(6):1951–64. doi: 10.1093/jxb/erw017 (PMC4783373; doi:10.1093/jxb/erw017)
Supplement: Supplementary Data [file supp_erw017_supplementary_tables_S1_S2_figures_S1_S9.pdf]

Type-*f* thioredoxins have a role in short-term activation of carbon metabolism and their loss affects growth under short-day conditions in *Arabidopsis thaliana*.

Belén Naranjo, Antonio Diaz-Espejo, Marika Lindahl, and Francisco Javier Cejudo

SUPPLEMENTARY DATA

Table S1. Sequence of oligonucleotides used for genotype analyses.

**Oligonucleotide**	**Sequence (5' - 3')**
Trx *f1* Fw	TTCATATGTGTAGCTTAGAAACCGTTAATG
Trx *f1* Rv	AAGTCGACAGAGACTGGTTCATCCG
Trx *f2* Fw	TGGTCCTTGCAAAGTGATTG
Trx *f2* Rv	CCCCGGTCACTTCCTTTACT
Trx *f2* RP	TTCTCAATTGCCAATTCTTGC
Trx *f2* LP	CTATTTCAACAATCGGGTCCC
Lba1	TGGTTCACGTAGTGGGCCATCG
o8409	ATATTGACCATCATACTCATTGC

Table S2. Sequence of oligonucleotides used for gene expression analyses.

**Oligonucleotide**	**Sequence (5’ - 3’)**
TRX f1 qFw	CGATGATCTGGTTGCAGCG
TRX f1 qRv	CTGGTTCATCCGGAAGCAG
TRX f2 qFw	TGTAACCAAGACAACAAGCCA
TRX f2 qRv	CGGTCACTTCCTTTACTACCT
TRX x qFw	TGCTTTCGTTAAGCTCCGGT
TRX x qRv	ATCTCTTTGATTCCGCCGCA
NtrC qFw	TGAAGATGAAGAAAGAGTACCGAG
NtrC qRv	GGTGTCCTCATTTATTGGCCT
Ubq qFw	GAAGTTCAATGTTTCGTTTCATGT
Ubq qRv	GGATTATACAAGGCCCCAAAA

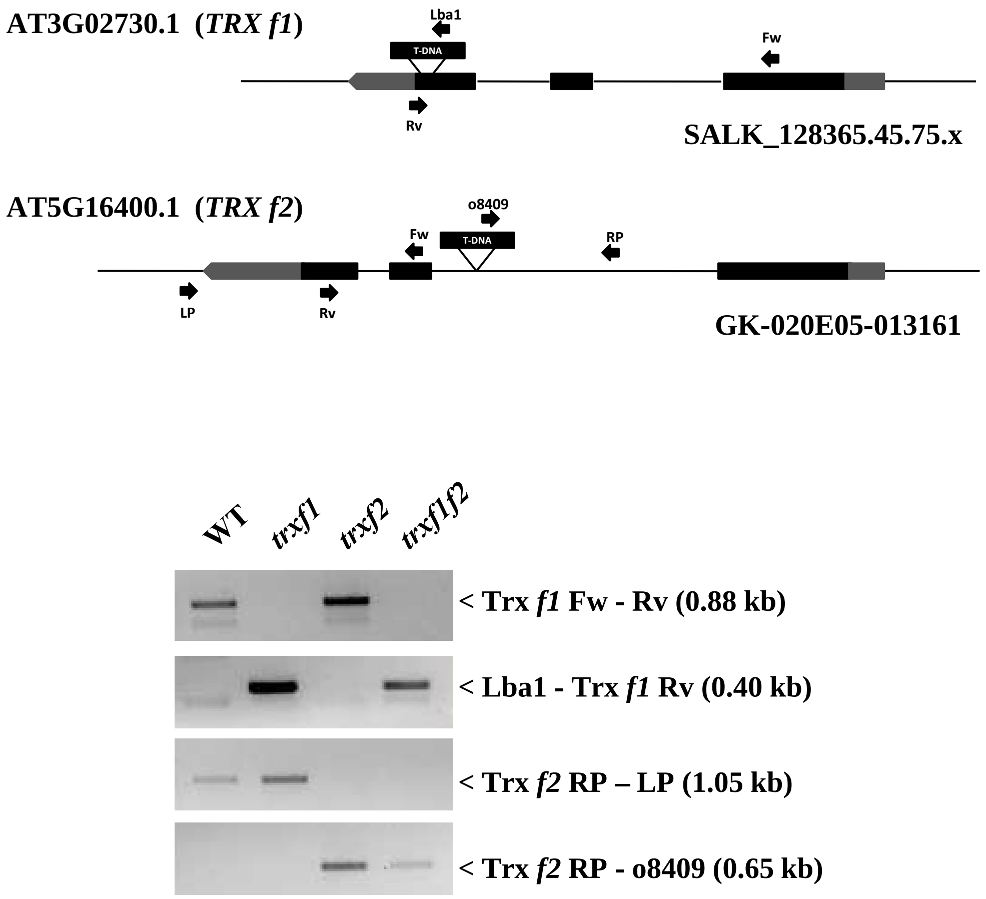

Figure S1. Genotype of Trx *f*-deficient mutants. The presence of the T-DNA insertions was tested by PCR using as template genomic DNA isolated from wild type and mutant plants (*trxf1*, *trxf2* and *trxf1f2*), as indicated. Band size and the pair of oligonucleotides of each reaction, as outlined in the scheme of both genes, are indicated on the right. Amplified DNAs were fractionated in 2% agarose gels and stained with ethidium bromide.

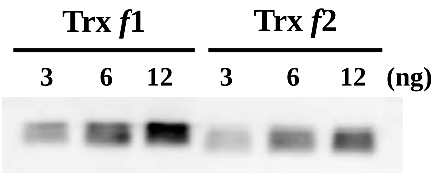

Figure S2. Cross-reaction of the anti Trx *f*1 antibody with Trx *f*1 and Trx *f*2. Trx *f*1 and Trx *f*2 proteins from *Arabidopsis,* containing a His-tag at the N-terminus, were expressed in *E. coli* and purified by nickel-affinity chromatography. Different amounts of both recombinant proteins were subjected to SDS-PAGE (15% polyacrylamide) under reducing conditions, transferred to nitrocellulose membrane and probed with anti-Trx *f*1 antibody (dilution 1/1000).

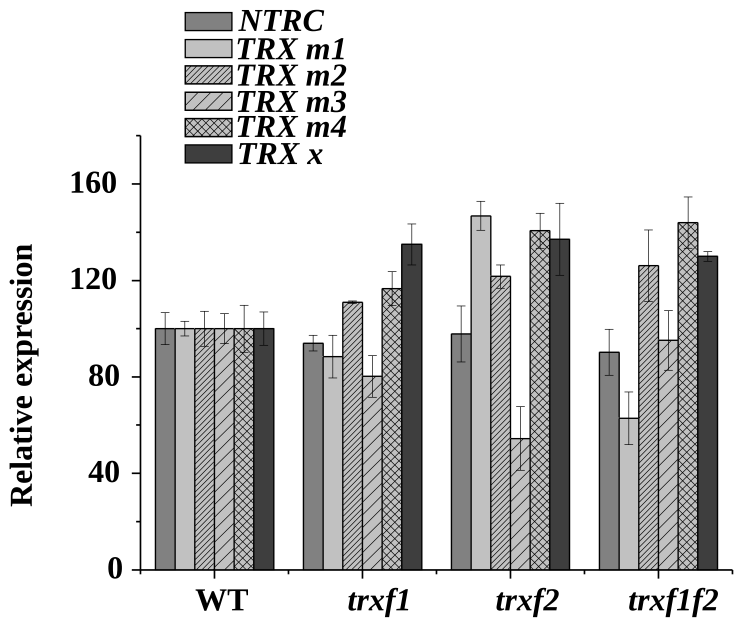

Figure S3. Level of *NTRC, m-* and *x*-type *TRX* gene transcripts in Trx *f*-deficient mutants. The content of transcripts of the different genes, as indicated, was determined by qRT-PCR. Total RNA was extracted from leaves of wild type, *trxf1*, *trxf2* and *trxf1f2* plants grown under long-day conditions during 22 days and harvested after 3 h illumination. The pairs of oligonucleotides used for cDNA amplification are indicated in Supplementary Table S2 except those of the *m*-type Trx genes, which were described in Barajas-López et al. (2011). Transcript levels were normalized to their level in wild type plants. Determinations were performed at least three times and mean values ± SD are represented.

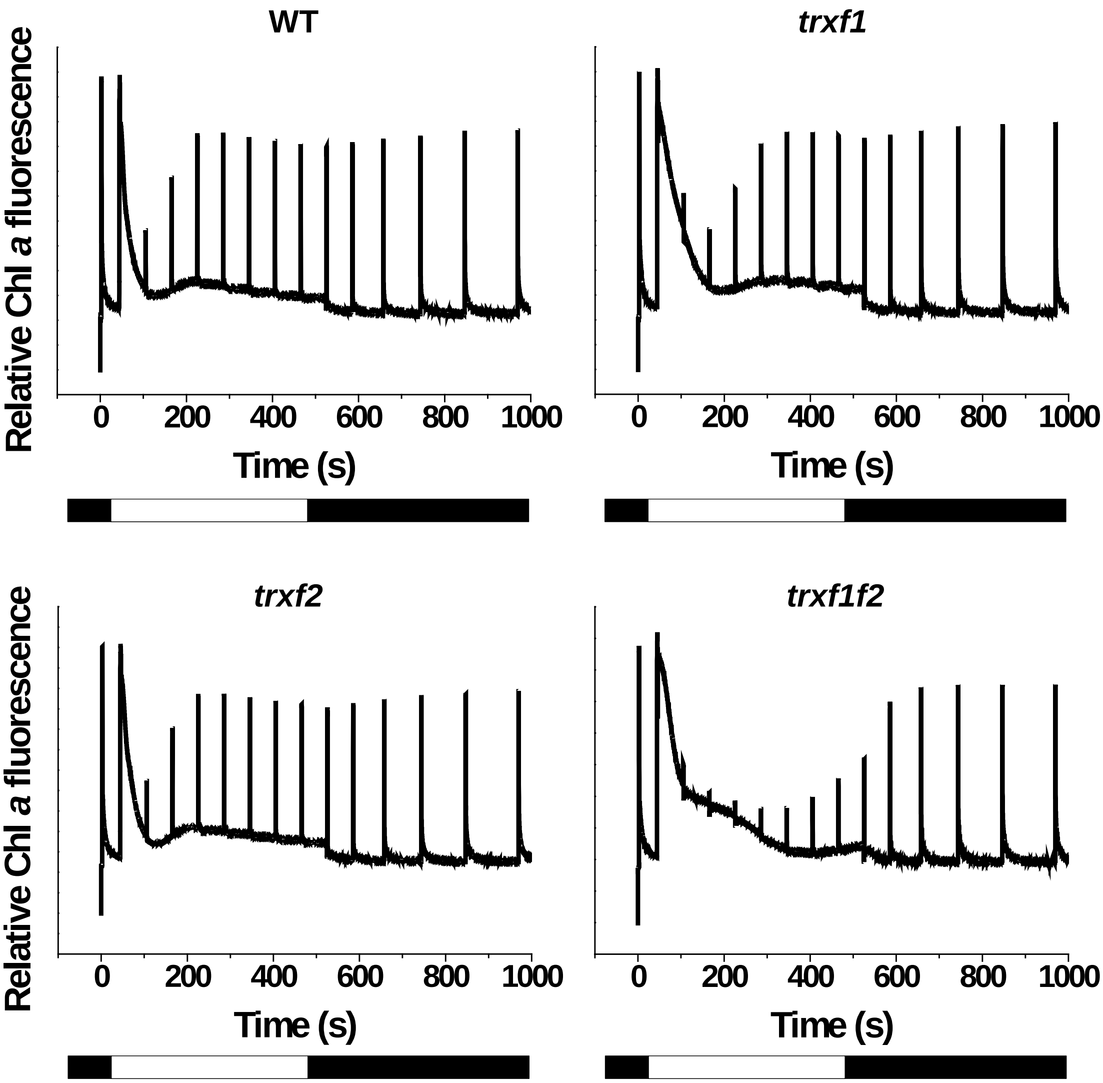

Figure S4. Chlorophyll *a* fluorescence of PSII. Representative fluorescence curves from the experiments presented in Fig. 5. Following 30 min dark adaptation and determination of F_0 and F_m, the actinic light was turned on and saturating pulses were applied every 60 s. Chlorophyll fluorescence was monitored during illumination with actinic light at 75 µE m^{-2} s^{-1} intensity followed by darkness. Light and dark periods are indicated by white and black bars, respectively.

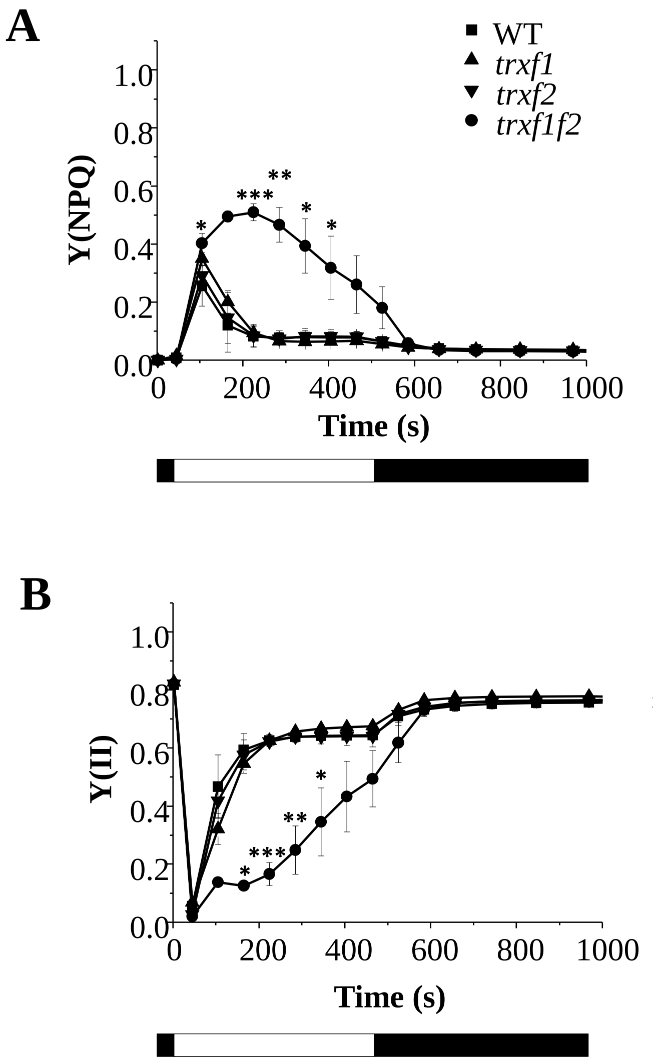

Figure S5. Photosystem II activity in wild type and Trx *f* deficient mutants grown under long-day conditions. Chlorophyll fluorescence of photosystem II (P680) was measured using a pulse-amplitude modulation fluorimeter. After incubation of plants, grown for 26 days under long-day conditions, during 30 min in darkness, an induction-recovery curve was performed to determine the quantum yields of non-photochemical quenching, Y(NPQ) (A), and photosystem II, Y(II) (B). During the 8 min induction period, a red (653 nm) actinic light at 75 µE m^{-2} s^{-1} intensity was applied. Thereafter, the actinic light was switched off and measurements were continued for another 10 min in the dark. Saturation pulses (10000 µE m^{-2} s^{-1}, 0.6 s) every 60 s were applied. Values are the mean±SD. Light and dark periods are indicated by white and black bars. Statistical significance (* $p < 0.05$; ** $p < 0.01$; *** $p < 0.001$) was determined with the Student's t test comparing values for each of the mutant lines to the wild type.

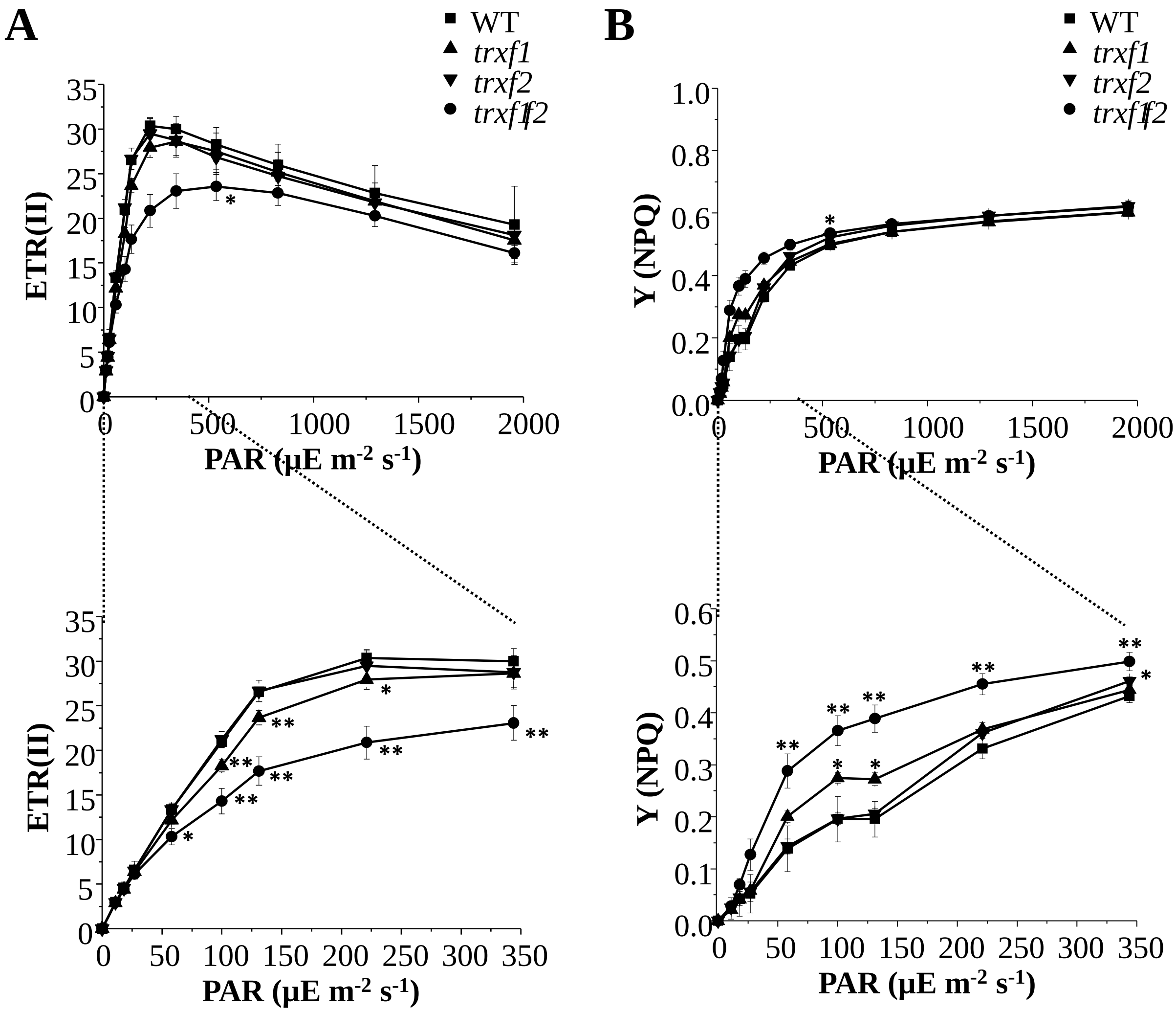

Figure S6. Light-dependent linear photosynthetic electron transport and NPQ in wild type and Trx *f* deficient mutants grown under long-day conditions.

A, Relative linear electron transport rates of photosystem II, ETR (II) was measured in pre-illuminated attached leaves of plants grown under long-day conditions at 125 µE m^{-2} s^{-1}. Chlorophyll fluorescence of photosystem II was determined using a pulse-amplitude modulation fluorimeter. Each data point is the mean ± SD. PAR (photosynthetically active radiation). B, Quantum yields of NPQ from the corresponding light-titration curves in A. Statistical significance (* $p < 0.05$; ** $p < 0.01$; *** $p < 0.001$) was determined with the Student's t test comparing values for each of the mutant lines to the wild type. Lower graphs show the same data but with expanded scale for lower PAR values.

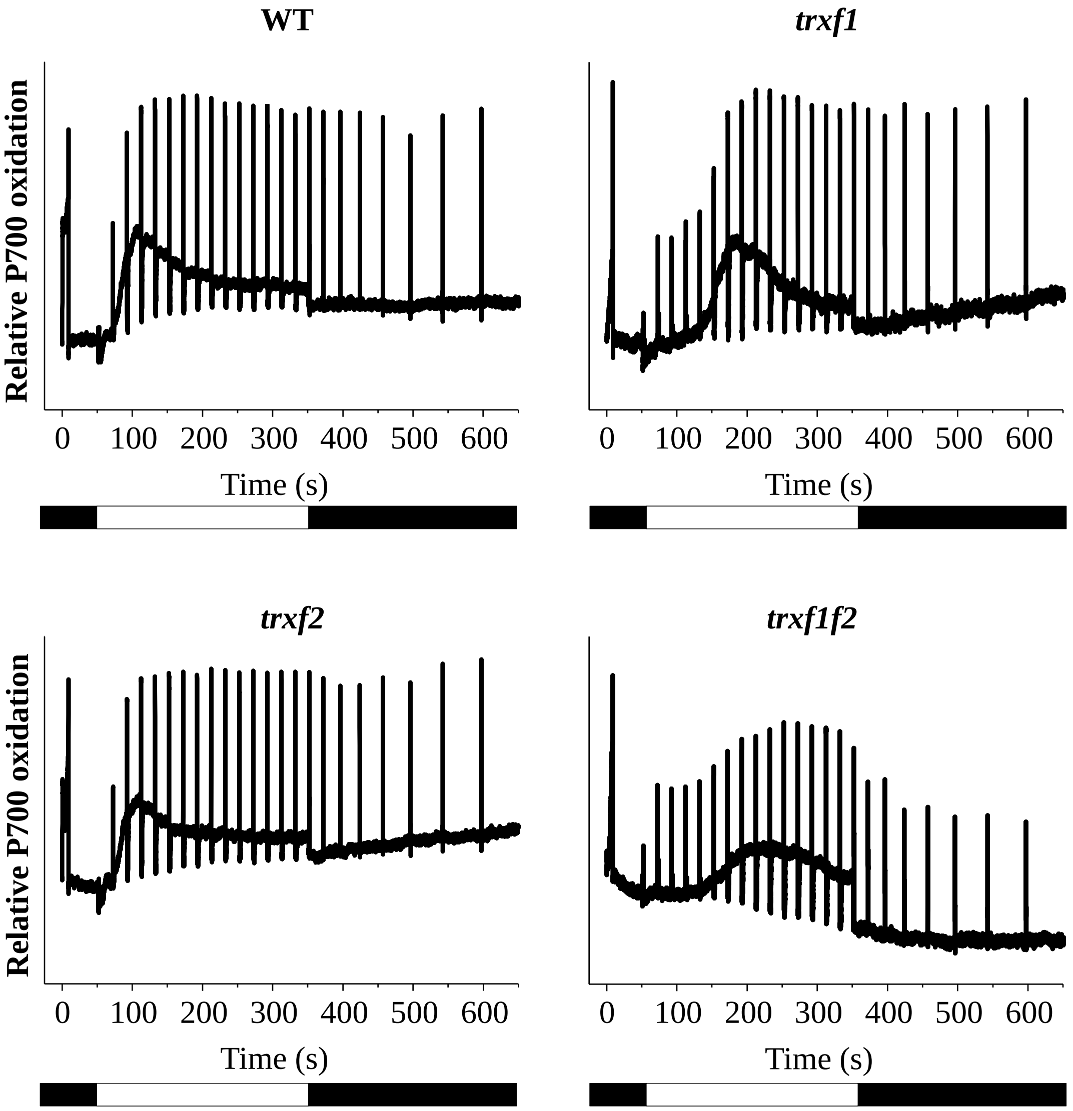

Figure S7. Absorbance of the oxidised form of photosystem I. Representative P700 absorbance at 830 nm from the experiments presented in Fig. 7. Following the initial determination of maximal oxidation of P700 under far red light, the actinic light at an intensity of 125 μE m^{-2} s^{-1} was switched on and saturating pulses were applied every 20 s. After 5 min, the light was switched off and measurements were continued for another 5 min.

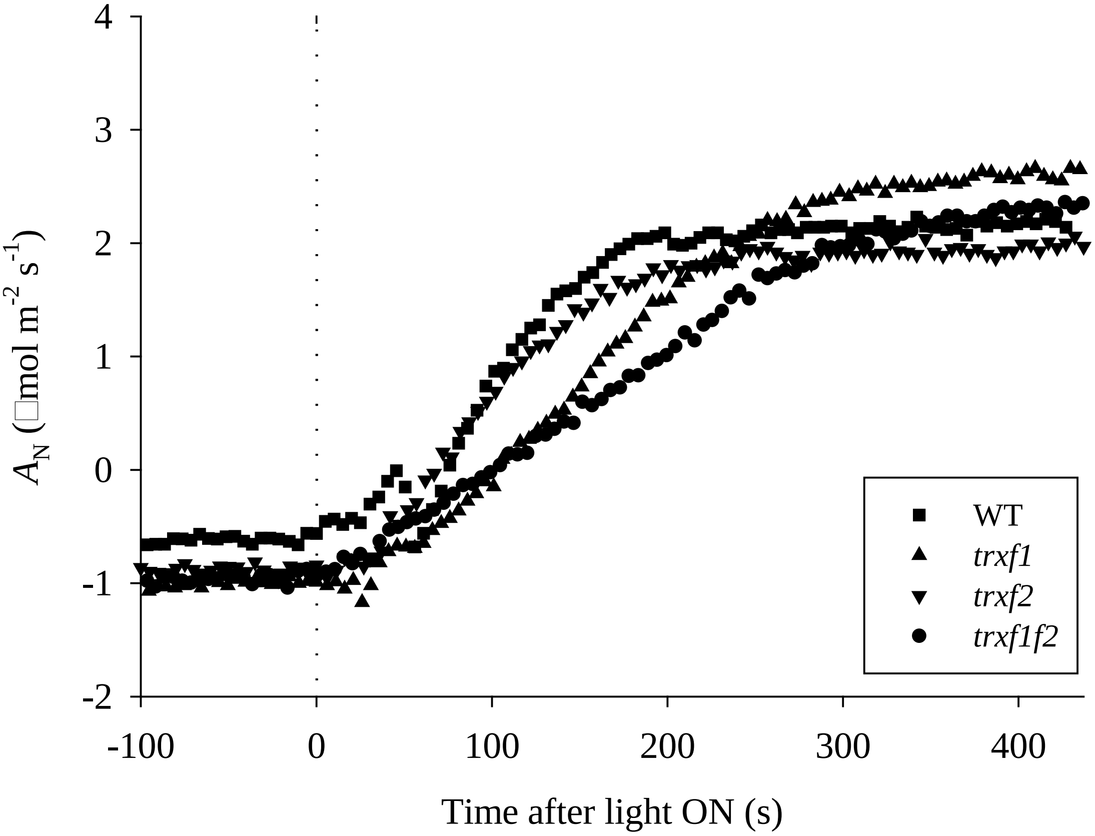

Figure S8. Net CO_2 assimilation rate (A_N) in wild type and Trx *f* deficient mutants. The rate of net CO_2 assimilation was determined with the aid of a portable IRGA Licor 6400 in short-day grown plants (50 days-old) that had been adapted to darkness. Vertical dashed line represents the time when light was turned on (75 µE m^{-2} s^{-1}). Six leaves per line were measured and one representative curve is shown for each line.

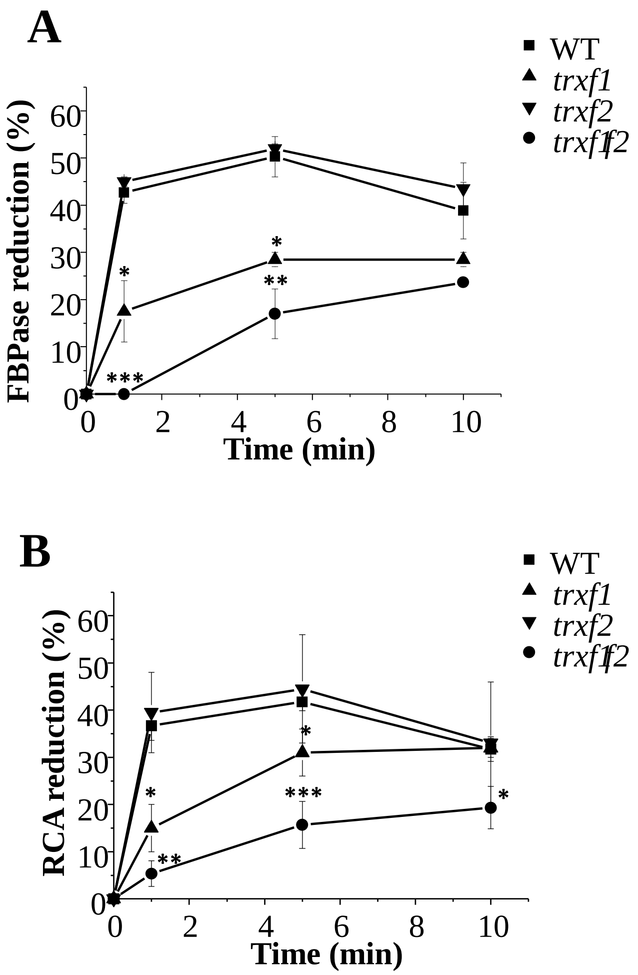

Figure S9. Light-dependent reduction FBPase and Rubisco activase. The redox state of FBPase and Rubisco activase (RCA) was determined by alkylation experiments followed by Western blot analyses. Samples from wild type and Trx *f*-deficient mutants were harvested at the end of the night (0 min) and after short exposures (1, 5 and 10 min) to growth light at 125 µE m^{-2} s^{-1} intensity. Band intensities were quantified and the percentage of reduced proteins was calculated as the ratio between the shifted band (reduced form) and the sum of reduced and oxidized forms. Each value is the mean of three independent experiments ± standard error (SE). Statistical significance (* $p < 0.05$; ** $p < 0.01$; *** $p < 0.001$) was determined with the Student's t test comparing values for each of the mutant lines to the wild type.
